# Provision of inguinal hernia surgery in first‐referral hospitals across low‐ and middle‐income countries: Secondary analysis of an international cohort study

**DOI:** 10.1002/wjs.12374

**Published:** 2024-11-22

**Authors:** Maria Picciochi, Philip Vareed Alexander, T. Anyomih, N. Boumas, R. Crawford, F. Enoch Gyamfi, N. Hopane, M. Isiagi, S. K. Kamarajah, V. Ledda, A. Matei, A. Mulliez, D. Nepogodiev, N. Roy, C. E. Okereke, R. Tubasiime, M. Steinruecke, A. Bhangu, NIHR Global Health Research Unit on Global Surgery

**Keywords:** global surgery, hernia

## Abstract

**Introduction:**

Surgical care in first‐referral hospitals (FRHs) in low‐ and middle‐income countries (LMICs) is poorly characterized. Inguinal hernia repair can act as a good tracer condition. This study aimed to evaluate the variation in hernia repair across different hospital types in LMICs.

**Methods:**

We conducted a secondary analysis of an international prospective cohort study of hernia surgery. Data was collected from consecutive patients undergoing primary inguinal hernia repair between 30 January and May 21, 2023. We characterized patients from LMICs, comparing first‐referral, secondary, and tertiary hospitals. Emergency surgery, financing methods, mesh use, and complications were defined as key performance measures relevant for FRHs. A multilevel logistic regression model was used to test associations between complications and hospital type.

**Results:**

This analysis included 8155 patients undergoing hernia repair across 328 hospitals in 55 LMICs. Most patients were male (89.8%, 7324/8155), of working age (mean age 41.6, SD 25.3). Emergency surgery rates were similar across first‐referral, secondary and tertiary hospitals (11.1%, 10.9%, and 9.6%, respectively). Patients in FRHs were most likely to experience out‐of‐pocket payments (31.4%, 9.4%, and 17.4%). They also had lower rates of mesh use (71.9%, 82.1%, and 84.1%) and higher postoperative complication rates (19.1%, 12.5%, and 14.0%), although complications were similar after adjustment (adjusted odds ratio 1.71, 95% CI 0.83–3.54, *p* = 0.148).

**Discussion:**

This sample of FRHs is capable of delivering simple elective surgery, reducing the burden on subsequent referral hospitals. To scale these surgical pathways, FRHs need support to increase the use of mesh and ensure cost protection for patients.

## 1 INTRODUCTION

First‐referral hospitals (FRHs) in low‐ and middle‐income countries (LMICs) have been essential in the delivery of surgical care for common surgical diseases to the most vulnerable populations.^1^ They are structured to be the first point of healthcare contact for rural populations.^2^, ^3^ Utilization varies with reports from the National Sample Survey Office 75^th^ Round (2017–2018) in India demonstrating that a significant percentage of the rural population first seeks healthcare from private practitioners or smaller clinics.^4^ A smaller percentage of patients used community health centers or first‐referral units. Nevertheless, FRHs remain the first easily accessible port of call for patients in rural areas. However, data to characterize the surgical care delivered in these settings are still scarce and needs better representation in global surgical studies.^5^

Inguinal hernia surgery is one of the most commonly performed operations worldwide with approximately 20 million repairs occurring every year.^6^ It is a low‐risk procedure, usually done as an elective case, and with low overall complication rates. Most of the burden of disease resides in LMICs,^7^ where it is performed by surgeons and nonsurgical physicians, usually through a groin incision and often under spinal or locoregional anesthesia.^8^ This procedure was included in the list of essential surgical procedures that should be delivered in FRHs.^2^

Whilst studying variation in all operations performed across FRHs is unlikely to be feasible, evaluating inguinal hernia repair can serve as a good marker of assessment of the elective surgical care across a wide range of hospitals.^9^ Comparing FRHs to secondary and tertiary hospitals can help identify differences in their populations and variation in the care that is delivered. To study the surgical care delivered by FRHs in LMICs, we conducted a secondary analysis of an international prospective cohort study of hernia surgery.^8^ This study used inguinal hernia repair as a tracer condition to compare access to and quality of elective care across different income groups.^8^ A secondary analysis using the same principle, taking hernia repair as a tracer condition, would allow a more comprehensive understanding of the variation between different hospital types in LMICs.

## 2 METHODS

### 2.1 Study objectives

The primary objective of this study was to compare surgery provided in first, second, and tertiary level hospitals, using a common tracer condition. The secondary objective was to better understand the characteristics of this FRH network, accepting that FRHs are very heterogenous, and those providing hernia surgery are likely to reflect one end of the spectrum. We aimed to explore characteristics in terms of hospital services provided and geographical area served.

### 2.2 Study design and participants

This was a secondary analysis of an international, prospective cohort study of patients undergoing inguinal hernia surgery,^8^ which compared a measurement set across different income groups. The study followed the principles of collaborative research and was disseminated and delivered by the NIHR Global Health Research Unit on Global Surgery Collaborative.^10^ Any hospital performing inguinal hernia repairs was eligible to take part. Consecutive patients undergoing primary inguinal hernia repair were included in 4‐week inclusion blocks between 30 January and May 21, 2023, regardless of age or urgency of surgery. Patients with an open approach via midline incision were excluded due to the higher complexity associated with these procedures.^11^ Only routine, anonymized data was collected and no changes were made to existing clinical care pathways. Ethical approval was obtained by the hospital lead, who was the local principal investigator, according to local regulations. Consent from patients was taken in some hospitals as required by the local research ethics committee, and other hospitals had a waiver from it.

### 2.3 Study setting

For this analysis, only patients operated in LMICs were included. The country income groups were defined by the World Bank in 2022 as upper‐middle income, lower‐middle income, and low‐income countries.^12^ We defined three levels of hospital based on the definitions used in previous global cohort studies^13^: (a) first‐referral, hospitals with few specialties, mainly internal medicine, obstetrics and gynecology, pediatrics, and general surgery; (b) secondary hospitals with 5 to 10 clinical specialties, usually with 200 to 800 beds; and (c) tertiary hospitals with highly specialized staff and technical equipment and clinical services highly differentiated by function. Hospitals were classified by the hospital lead in the hospital questionnaire which was completed by the hospital lead at each site and verified by a consultant to ensure data quality. This was done in parallel to patient data collection and allowed for a better characterization of the hospitals taking part in the study. The data management plan was the same as described in the main analysis.^8^

### 2.4 Hospitals coverage

For each hospital, GPS coordinates and location were identified. Population density was calculated within a 5‐km radius of each hospital using an existing online tool (https://tomforth.co.uk/circlepopulations/). This distance was selected because it represents a walking distance considering that not all hospitals are easily accessible by road and it was previously used in studies measuring access to a healthcare facility.^14^ We accepted a prior that is only one of many possible geospatial characteristics, and so we considered this an exploratory study. Comparison between the population densities covered by each hospital type was presented to further characterize each participating hospital.

### 2.5 Measurement set

The measurement set was previously developed and tested to evaluate the attributes of the World Health Organization's Health System Building Blocks.^8^ The measurement set evaluated access and coverage, quality, and safety. The writing group, which included a network of surgeons working in FRHs in LMICs, selected four key performance measures that were most relevant for FRHs.^15^ These were emergency rate, financing methods, mesh use, and postoperative complications. Among the financing methods, out‐of‐pocket expenditure was selected as the most relevant, considering the relationship with patients' vulnerability.^16^ The other measures were considered additional descriptive data (Supplementary Table 1).

Each measure was applied to a pre‐specified set of patients to ensure relevance and to be compliant with the recommendations.^17^, ^18^ Urgency of surgery, bowel resection rates, and postoperative complications were assessed in all patients. Postoperative complications were evaluated at 30 days after surgery, either in person, by phone, or by checking the health records, without any changes to their routine follow‐up. Waiting times were measured among patients who underwent elective repair. Mesh use was assessed among adults who underwent elective repair. Day‐case surgery was measured among adults undergoing elective repair with an ASA of I–II and age younger than 90 years, which is the group where day‐case surgery was recommended to be adopted. The other measures were assessed in all patients and hospitals and compared between the three hospital levels.

### 2.6 Statistical analysis

Continuous variables with a normal distribution were presented as mean and standard deviation, and skewed variables were presented as median and interquartile range. Categorical variables were reported with frequencies and percentages. Adjusted rates of key performance measures for each type of hospital were derived using a multilevel logistic regression model and presented with 95% confidence intervals. Hospitals nested within countries were included as random effects. The rates of additional descriptive measures were presented as unadjusted rates. All statistical analyses were performed using R (version 4.0.2, R Foundation for Statistical Computing, Vienna, Austria).

### 2.7 Role of the funder

The funders of the study had no role in study design, data collection, data analysis, data interpretation, or writing of the report.

## 3 RESULTS

**FIGURE 1:**
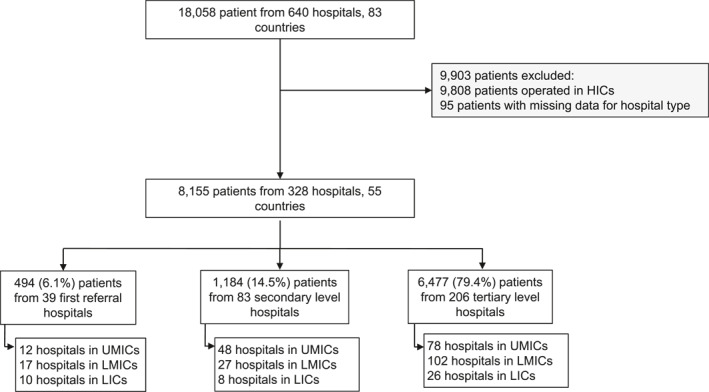
Flowchart of included patients. Data are n (%) unless stated otherwise. HICs: high income countries, UMICs: upper‐middle income countries, LMICs: lower‐middle income countries, LICs: low‐income countries.

Overall, data was collected from 18,058 patients undergoing inguinal hernia repair in 83 countries. After excluding missing data (*n* = 95) and patients from HIC countries (*n* = 9808), this analysis included 8155 patients from 328 hospitals in 55 upper‐middle‐, lower‐middle‐, and low‐income countries. This included 39 FRHs, 83 secondary hospitals, and 206 tertiary hospitals, as shown in Figure 1 and comparisons were made in this order.

**FIGURE 2:**
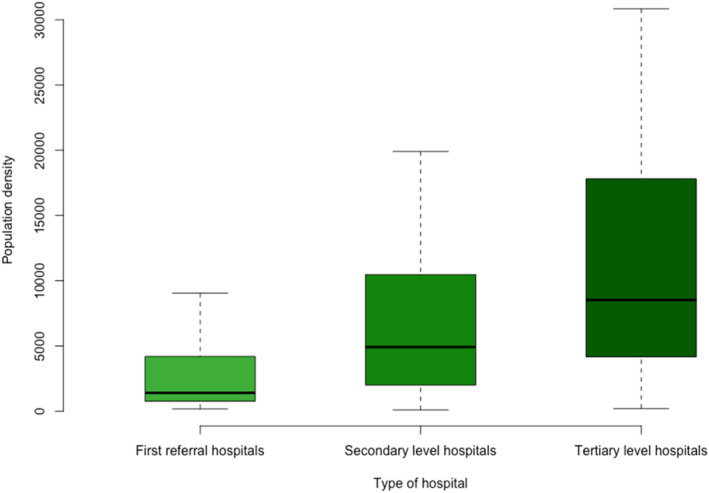
5 km population density presented on patients treated by hospital type.

Most patients underwent surgery in publicly funded hospitals (79.9%, 6513/8155), independent of hospital type, as shown in Table 1. However, privately funded hospitals had the highest representation among FRHs, when compared to secondary and tertiary hospitals (38.5%, 25.3%, and 17.0%, respectively, Supplementary Table 2). Emergency surgery was available during 24 h in most hospitals (89.9%, 295/328), but there were four FRHs where it was not available at any time of the day (10.3%, 4/39). The median number of beds increased from first‐referral to tertiary hospitals (100.0, 250.0, and 750.0, respectively). Population density within a 5‐km radius of each hospital, weighted with the patients involved in the study per hospital, was lower among FRHs (median 1398.5 per sqkm), as shown in Figure 2.

**TABLE 1:** Hospital characteristics and variation.

	First‐referral hospitals (*n* = 494)	Secondary level hospitals (*n* = 1184)	Tertiary level hospitals (*n* = 6477)	Total (*n* = 8155)
Income group				
UMIC	129 (26.1%)	598 (50.5%)	2572 (39.7%)	3299 (40.5%)
LMIC	201 (40.7%)	490 (41.4%)	3257 (50.3%)	3948 (48.4%)
LIC	164 (33.2%)	96 (8.1%)	648 (10.0%)	908 (11.1%)
(Missing)	0	0	0	0
Hospital funding				
Public	296 (59.9%)	883 (74.6%)	5334 (82.4%)	6513 (79.9%)
Private	165 (33.4%)	289 (24.4%)	952 (14.7%)	1406 (17.2%)
Public‐private	33 (6.7%)	12 (1.0%)	191 (2.9%)	236 (2.9%)
(Missing)	0	0	0	0
Surgical emergency service				
Yes ‐ surgery available 24h	361 (73.1%)	986 (83.3%)	6107 (94.3%)	7454 (91.4%)
Yes ‐ surgery only during daytime	37 (7.5%)	95 (8.0%)	233 (3.6%)	365 (4.5%)
Yes ‐ for assessment only	43 (8.7%)	103 (8.7%)	56 (0.9%)	202 (2.5%)
No	53 (10.7%)	0 (0.0%)	81 (1.3%)	134 (1.6%)
(Missing)	0	0	0	0
Number of beds				
Median (IQR)	100.0 (80.0–103.0)	250.0 (200.0–413.0)	750.0 (435.0–1200)	550.0 (350.0–1047)

*Note*: Data are *n* (%) unless stated otherwise.

Abbreviations: HIC: high‐income country; LIC: low‐income country; LMIC: lower‐middle‐income country; UMIC: upper‐middle‐income country.

Patients undergoing hernia repair in LMICs were mostly male (89.8%, 7324/8155) and symptomatic (80.3%, 6549/8155). Most patients were of working age (mean age 41.6, SD 25.3) and had low ASA scores (94.1%, 7670/8155). FRHs operated a higher proportion of large hernias, including those extending to the scrotum, mid‐thigh, and beyond (40.5%, 31.4%, and 33.3% respectively). Most operations were clean (93.4%, 7620/8155) and the hernia defect size varied across all hospitals, as shown in Table 2.

**TABLE 2:** Included patients.

	First‐referral hospitals (*n* = 494)	Secondary level hospitals (*n* = 1184)	Tertiary level hospitals (*n* = 6477)	Total (*n* = 8155)
Age				
Mean (SD)	41.2 (23.3)	43.7 (24.3)	41.3 (25.6)	41.6 (25.3)
Sex				
Female	49 (9.9%)	144 (12.2%)	637 (9.8%)	830 (10.2%)
Male	445 (90.1%)	1040 (87.8%)	5839 (90.2%)	7324 (89.8%)
(Missing)	0	0	0	0
ASA grades				
ASA I‐II	478 (96.8%)	1119 (94.5%)	6073 (93.8%)	7670 (94.1%)
ASA III‐V	14 (2.8%)	63 (5.3%)	384 (5.9%)	461 (5.7%)
Not recorded	2 (0.4%)	2 (0.2%)	19 (0.3%)	23 (0.3%)
(Missing)	0 (0.0%)	0 (0.0%)	1 (0.0%)	1 (0.0%)
Symptoms				
Asymptomatic	54 (10.9%)	184 (15.5%)	1367 (21.1%)	1605 (19.7%)
Symptomatic	440 (89.1%)	1000 (84.5%)	5109 (78.9%)	6549 (80.3%)
(Missing)	0	0	0	0
Hernia size				
Limited to inguinal region	294 (59.5%)	812 (68.6%)	4319 (66.7%)	5425 (66.5%)
Limited to scrotum	193 (39.1%)	354 (29.9%)	2069 (31.9%)	2616 (32.1%)
Extend to mid‐thigh or beyond	7 (1.4%)	18 (1.5%)	88 (1.4%)	113 (1.4%)
(Missing)	0	0	1	1
Contamination				
Clean	484 (98.0%)	1124 (94.9%)	6012 (92.8%)	7620 (93.4%)
Clean‐contaminated	484 (98.0%)	1124 (94.9%)	6012 (92.8%)	7620 (93.4%)
Contaminated	0 (0.0%)	7 (0.6%)	28 (0.4%)	35 (0.4%)
Dirty	0 (0.0%)	1 (0.1%)	11 (0.2%)	12 (0.1%)
(Missing%)	0	1	2	3
Hernia defect size				
<1.5 cm	76 (15.4%)	231 (19.5%)	1605 (24.8%)	1912 (23.5%)
1.5–3 cm	173 (35.0%)	447 (37.8%)	2378 (36.7%)	2998 (36.8%)
>3 cm	212 (42.9%)	408 (34.5%)	1744 (26.9%)	2364 (29.0%)
Not known	33 (6.7%)	97 (8.2%)	749 (11.6%)	879 (10.8%)
(Missing)	0	0	0	0

*Note*: Data are *n* (%) unless stated otherwise. ASA: American Society of Anesthesiologists Physical Status Classification System grade.

**FIGURE 3:**
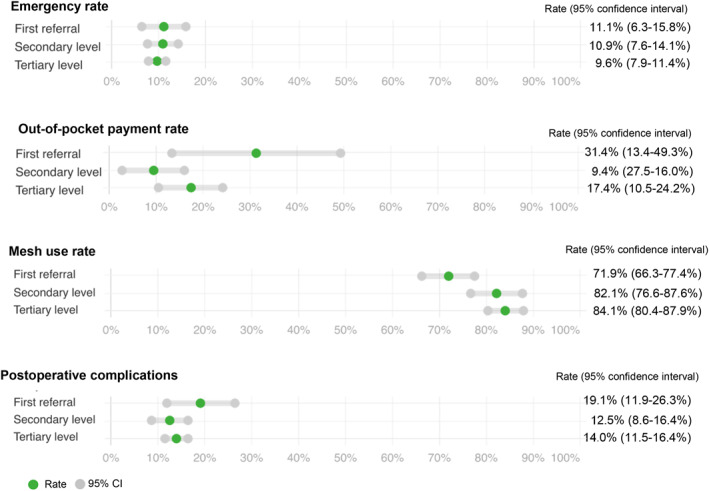
Key performance measures.

Regarding the key performance measures relating to access, emergency surgery rates showed little variation across hospital types (adjusted rates 11.1%, 10.9%, and 9.6%). Overall, insurance by the government was the most common payment method (67.2%, 5479/8155, Supplementary Table 3). The out‐of‐pocket payment rates were highest in FRHs (31.4%, 9.4%, and 17.4%), as shown in Figure 3.

Regarding the key performance measures relating to quality, mesh use in adults undergoing elective repair increased from FRHs to tertiary hospitals (adjusted rates of 71.9%, 82.1%, and 84.1%). Postoperative complications at 30 days varied between hospitals, with the highest rates in FRHs (19.1%, 12.5%, and 14.0%). Most of these were minor complications (Clavien Dindo I–II 14.0%, 1137/8155, Supplementary Table 4). A multilevel logistic regression model did not identify significant differences in the complication rate by hospital type (adjusted odds ratio for FRHs 1.71, 95% CI 0.83–3.54, *p*‐value 0.1483).

The additional descriptive data evaluating access and coverage showed that bowel resection rates were similar among different hospital types (2.4%, 1.6%, and 2.5%). The variation in waiting times for elective repair was small (median 8 months in FRHs and 6 months in secondary and tertiary hospitals). Standardized patient pathways, waiting list management, and day‐case surgical units were available equitably across the different hospital types, as shown in Table 3.

**TABLE 3:** Additional descriptive data to measure access across the hospital types.

	First‐referral hospitals (*n* = 494)	Secondary level hospitals (*n* = 1184)	Tertiary level hospitals (*n* = 6477)	Total (*n* = 8155)
ACCESS AND COVERAGE				
Bowel resection				
Yes	12 (2.4%)	19 (1.6%)	159 (2.5%)	190 (2.3%)
No	482 (97.6%)	1163 (98.4%)	6316 (97.5%)	7961 (97.7%)
(Missing)	0	2	2	4
Total	*n* = 442	*n* = 1053	*n* = 5920	*n* = 7415
Waiting time to elective surgery				
Median (IQR)	8.0 (2.3–24.0)	6.0 (3.0–16.4)	6.0 (2.6–18.0)	6.0 (2.7–18.0)
(Missing)				
Waiting time before diagnosis				
Median (IQR)	6.1 (1.9–18.1)	4.3 (1.5–11.7)	4.0 (1.2–11.9)	4.0 (1.3–11.9)
(Missing)				
Waiting time after diagnosis				
Median (IQR)	0.8 (0.2–3.0)	1.9 (0.5–5.7)	2.1 (0.7–6.0)	2.0 (0.6–6.0)
(Missing)				
Waiting time after decision for surgery				
Median (IQR)	0.2 (0.1–0.8)	0.6 (0.2–1.6)	0.7 (0.1–2.0)	0.6 (0.1–1.8)
(Missing)				
Total	*n* = 368	*n* = 853	*n* = 4559	*n* = 5780
Standardized patient pathways				
Yes	273 (55.3%)	690 (58.3%)	3770 (58.2%)	4733 (58.0%)
No	221 (44.7%)	494 (41.7%)	2707 (41.8%)	3422 (42.0%)
(Missing)	0	0	0	0
Waiting list management				
Yes	431 (87.2%)	1040 (87.8%)	5580 (86.2%)	7051 (86.5%)
No	63 (12.8%)	144 (12.2%)	897 (13.8%)	1104 (13.5%)
(Missing)	0	0	0	0
Availability of day case surgical unit				
Yes	361 (73.1%)	1001 (84.5%)	5019 (77.5%)	6381 (78.2%)
No	133 (26.9%)	183 (15.5%)	1458 (22.5%)	1774 (21.8%)
(Missing)	0	0	0	0

*Note*: This table shows the variation of additional descriptive data to evaluate access across hospital types.

**FIGURE 4:**
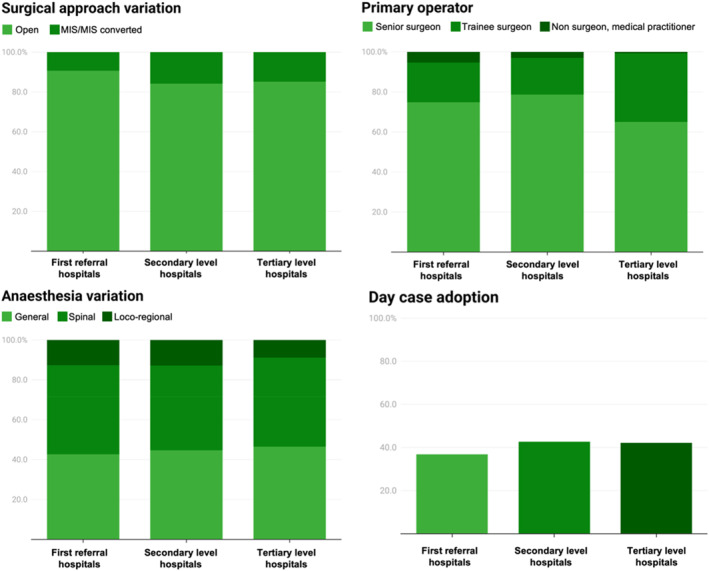
Additional descriptive data measuring quality across the hospital levels.

The additional descriptive data evaluating quality and safety varied between hospital types. In all hospitals, most patients were operated by a senior surgeon (67.6%, 5509/8155). However, differences were noted in the use of nonsurgical medical practitioners. In tertiary hospitals, they rarely performed operations (0.7%, 43/6477), as opposed to the FRHs, where it had a higher rate (5.3%, 26/494). Anesthesia type varied between the three hospital levels. General and spinal anesthesia were commonly adopted with differences of less than 2% between the different hospital types. The main difference was the use of locoregional anesthesia, which had higher adoption in FRHs and secondary hospitals (12.6%, 12.8%, and 8.7%, respectively). Minimally invasive surgery had limited adoption in all hospitals, but FRHs showed the lowest rate (9.3%, 46/494). Day‐case surgery was adopted in less than half of the eligible patients across all hospitals (41.9%, 2263/5399), as shown in Figure 4 and Supplementary Table 4.

## 4 DISCUSSION

This study highlights key differences and similarities between patients undergoing hernia surgery in first‐referral, secondary, and tertiary level hospitals in LMICs. It shows that the FRHs represented in this study are likely to have similar complication rates to tertiary centers, but patients are less likely to have mesh placed. While mesh availability was already known to be lower in LMICs, the variation across different hospital types suggests that this particularly affects FRHs. The remote location of these hospitals might account for the challenges in delivering mesh to these areas.^19^ The cost of the mesh would also impact the utility and availability of mesh in rural populations.^20^

The other important finding was that patients operated in FRHs were most likely to have out‐of‐pocket expenditures. This highlights the vulnerability of this population, who required more financial protection to access surgical care and were then less likely to receive a repair with mesh, which compounds their lack of access to it. Considering the high levels of poverty in the regions covered by FRHs, this finding identifies the susceptibility of these patients to catastrophic expenditures.^3^ High out‐of‐pocket payments in financially vulnerable populations may also set the stage for delayed presentations with larger hernias that extend to the scrotum, midthigh, and beyond in FRHs.^21^ The higher representation of privately funded hospitals in this category might partly explain this observed difference.^22^ This funding and financing mechanism seems to be able to support elective surgery but has less capacity to deliver emergency surgery and is only available to a restricted group of patients, who were able to fund their care.

This study also showed that patients undergoing hernia repair in FRHs were most likely to be operated on by nonsurgical medical practitioners. The lack of surgeons or nonsurgical practitioners trained in using mesh for hernia repair might play a role.^23^ A lack of surgeons in these settings might be a reason for the higher use of nonsurgical medical practitioners.^1^

There are limitations to this study. First, the FRHs represented in this study are all capable of providing hernia repair and most likely represent one end of the spectrum of these types of hospitals; that is, they are probably the larger and better resourced of the FRHs. However, they represent a large number across multiple countries and so have provided real‐world patient data rather than survey data alone. Second, there might be differences in FRHs from upper‐middle‐, lower‐middle‐, and low‐income countries which were not fully explored in this study, given the low number of patients included. Third, this study was not intended to be a comprehensive evaluation of all FRHs but rather a purposeful selection of hospitals identified by invitation, subscription, and snowball methods of dissemination. The higher proportion of privately funded FRH than expected might be a marker of that.^24^ Finally, the fact that not all patients had a routine follow‐up appointment at 30 days after surgery might have underestimated the complication rates. However, this is not possible in all settings and the strategy followed had likely captured all patients with major events who would represent for further care in their hospitals.

The findings from this study identified different areas for intervention to improve access to and quality of surgical care in FRHs. The analysis that was conducted and focused on different hospital levels all based in LMICs allowed a better understanding of the variation present there, which was not explored before. Hernia surgery might be safely expanded in FRHs, but access to mesh can be improved. As supported by previous evidence, this will decrease recurrence rates and the need for further surgery.^17^, ^25^ Considering that hernia repair is a simple and low‐risk procedure, improving capacity and training in FRHs will be better for their populations since these hospitals tend to be closer to local communities.^26^ At the same time, populations covered by FRHs need to have access to better payment mechanisms and increasing insurance by governments could be part of a solution.^24^

Future research to fully understand and characterize surgical care in FRHs is still needed. An established network of surgeons from FRHs will be essential to identify and recruit more centers in future studies. A better understanding of outcomes of patients who sought care in FRHs but ended up having an operation in secondary or tertiary level hospitals is relevant, but it was beyond the scope of this study. Exploring it further will identify the capacity limits within FRHs. It is important to identify which procedures in the essential surgery package can be delivered safely in FRHs and which should be referred to secondary and tertiary hospitals.^2^ There might be conditions where this varies across health systems, but a thorough review of these procedures would be beneficial.^27^ These procedures could then be used subsequently as tracers to evaluate healthcare in these populations. Building surgical capacity and accessibility to surgical care in remote populations will be the key to addressing the problem of the missing millions of surgical procedures that need to be performed in rural populations but are not being done currently.

## AUTHOR CONTRIBUTIONS

**Maria Picciochi**: Conceptualization; Data curation; Formal analysis; Funding acquisition; Investigation; Methodology; Project administration; Resources; Software; Visualization; Writing–original draft; Writing–review & editing. **Philip Vareed Alexander**: Conceptualization; Investigation; Methodology; Supervision; Writing–review & editing. **NIHR Global Health Research Unit on Global Surgery**: (Study management group: conceptualisation, methodology, investigation, resources, data curation, project administration and funding acquisition, Data handling and management: validation, investigation, resources and project administration, Dissemination Committee: conceptualisation, investigation, resources, data curation, and project administration, Hospital leads: validation, investigation, resources, data curation and project administration, Collaborators: investigation, resources, data curation and project administration).

## CONFLICT OF INTEREST STATEMENT

None.

## DOI STATEMENT

Maria Picciochi reports a project research grant from Portuguese Hernia and Abdominal Wall Society (Sociedade Portuguesa de Hernia e Parede Abdominal). Aneel Bhangu reports a Global Health Research Unit grant from the National Institute for Health Research (NIHR).

## ETHICS STATEMENT

Ethical approval was obtained by the hospital lead, according to local regulations.

## Supporting information

Supplementary Material

## Data Availability

Anonymized data are available upon request from the writing group and successful completion of a data sharing agreement through an Application Programming Interface linked to the REDCap data server hosted at University of Birmingham, Birmingham, UK.

## References

[1] Jeffries, Mazhar R. , T. M. Willows , S. Bhattarai , C. S. Tinn , N. Misago , and M. English . Feb 22 2024. “First Referral Hospitals in Low‐ and Middle‐Income Countries: the Need for a Renewed Focus.” Health Policy and Planning 39(2): 224–232. 10.1093/heapol/czad120. 38386923 PMC11031140

[2] Mock, C. N. , P. Donkor , A. Gawande , D. T. Jamison , M. E. Kruk , and H. T. Debas . 2015. “Essential Surgery: Key Messages of This Volume.” In Essential Surgery: Disease Control Priorities, Third Edition (Volume 1), edited by H. T. Debas , P. Donkor , A. Gawande , D. T. Jamison , M. E. Kruk and C. N. Mock . 26740991

[3] Meara, John G. , Andrew J. M. Leather , Lars Hagander , Blake C. Alkire , Nivaldo Alonso , Emmanuel A. Ameh , Stephen W. Bickler , et al. Aug 8 2015. “Global Surgery 2030: Evidence and Solutions for Achieving Health, Welfare, and Economic Development.” Lancet 386(9993): 569–624. 10.1016/S0140-6736(15)60160-X. 25924834

[4] India MoSPIGo . National Sample Survey Office (NSSO) 75th Round (2017‐2018) 2018. Accessed 9/07/2024. https://microdata.gov.in/nada43/index.php/catalog/152

[5] English, Mike , Laetitia Rispel , Freddie Ssengooba , and Nigel Edwards . Mar 2024. “Breaking the Silence on First Referral Hospitals and Universal Health Coverage.” Lancet Global Health 12(3): e366–e367. 10.1016/S2214-109X(23)00589-2. 38365407

[6] Köckerling, Ferdinand , and Maarten P. Simons . Apr 2018. “Current Concepts of Inguinal Hernia Repair.” Visceral Medicine 34(2): 145–150. 10.1159/000487278. 29888245 PMC5981671

[7] Ma, Qiuyue , Wenzhan Jing , Xiaoli Liu , Jue Liu , Min Liu , and Jie Chen . Mar 1 2023. “The Global, Regional, and National Burden and its Trends of Inguinal, Femoral, and Abdominal Hernia from 1990 to 2019: Findings from the 2019 Global Burden of Disease Study ‐ a Cross‐Sectional Study.” International Journal of Surgery 109(3): 333–342. 10.1097/JS9.0000000000000217. 37093073 PMC10389329

[8] NIHR Global Health Research Unit on Global Surgery . May 23 2024. “Access to and Quality of Elective Care: a Prospective Cohort Study Using Hernia Surgery as a Tracer Condition in 83 Countries.” Lancet Global Health. 10.1016/S2214-109X(24)00142-6. 38797188

[9] Naluyimbazi, Rovine , and Tamara N. Fitzgerald . Jul 2024. “Hernia Repair as a Tracer for Elective Surgical Care.” Lancet Global Health 12(7): e1069–e1070. 10.1016/S2214-109X(24)00214-6. 38797189

[10] Li, Elizabeth , and Aneel Bhangu . Jun 14 2022. “Collaborative Research in Surgery: a Rising Tide Lifts All Boats.” British Journal of Surgery 109(7): 576–577. 10.1093/bjs/znac099. 35416927 PMC10364717

[11] Picciochi, Maria , Daoud Chaudhry , Muhammed Elhadi , Ieva Jakaityte , Setthasorn Zhi Yang Ooi , Adesoji Ademuyiwa , Adewale Adisa , et al. 2024. “Protocol for a Global Cohort Study: HernIas, Pathway and Planetary Outcomes for Inguinal Hernia Surgery (HIPPO). Protocol.” Impact Surgery 1(3): 145–153. 10.62463/surgery.51.

[12] World Bank group . 2022. “World Bank Lis of Economies.”. https://datahelpdesk.worldbank.org/knowledgebase/articles/906519‐world‐bank‐country‐and‐lending‐groups.

[13] COVIDSurg Collaborative, GlobalSurg Collaborative . Jun 2021. “Timing of Surgery Following SARS‐CoV‐2 Infection: an International Prospective Cohort Study.” Anaesthesia 76(6): 748–758. 10.1111/anae.15458. 33690889 PMC8206995

[14] Hanson, Claudia , Jonathan Cox , Godfrey Mbaruku , Fatuma Manzi , Sabine Gabrysch , David Schellenberg , Marcel Tanner , Carine Ronsmans , and Joanna Schellenberg . Jul 2015. “Maternal Mortality and Distance to Facility‐Based Obstetric Care in Rural Southern Tanzania: a Secondary Analysis of Cross‐Sectional Census Data in 226 000 Households.” Lancet Global Health 3(7): e387–e395. 10.1016/S2214-109X(15)00048-0. 26004775

[15] Alexander, Philip . 2024. “Global Surgery in Rural Settings: Where Are the Giants?” Impact Surgery 1(3): 78. 10.62463/surgery.56.

[16] Schokkaert, Erik , Jonas Steel , and Carine Van de Voorde . Oct 2017. “Out‐of‐Pocket Payments and Subjective Unmet Need of Healthcare.” Applied Health Economics and Health Policy 15(5): 545–555. 10.1007/s40258-017-0331-0. 28432643

[17] The HerniaSurge Group . Feb 2018. “International Guidelines for Groin Hernia Management.” Hernia 22(1): 1–165. 10.1007/s10029-017-1668-x. PMC5809582 29330835

[18] Stabilini, Cesare , Nadine van Veenendaal , Eske Aasvang , Ferdinando Agresta , Theo Aufenacker , Frederik Berrevoet , Ine Burgmans , et al. Sep 5 2023. “Update of the International HerniaSurge Guidelines for Groin Hernia Management.” BJS Open 7(5). 10.1093/bjsopen/zrad080. PMC10588975 37862616

[19] Rajbhandari, Ruma , Devon E. McMahon , Joseph J. Rhatigan , and Paul E. Farmer . Jan 30 2020. “The Neglected Hospital ‐ the District Hospital's Central Role in Global Health Care Delivery.” New England Journal of Medicine 382(5): 397–400. 10.1056/NEJMp1911298. 31995684

[20] NIHR Global Health Research Unit on Global Surgery . Jul 2 2024. “Global Access to Technologies to Support Safe and Effective Inguinal Hernia Surgery: Prospective, International Cohort Study.” British Journal of Surgery(7): 111. 10.1093/bjs/znae164. PMC11235323 38985889

[21] Kagaigai, Alphoncina , Amani Anaeli , Sverre Grepperud , and Amani Thomas Mori . Aug 17 2023. “Healthcare Utilization and Catastrophic Health Expenditure in Rural Tanzania: Does Voluntary Health Insurance Matter?” BMC Public Health 23(1): 1567. 10.1186/s12889-023-16509-7. 37592242 PMC10436390

[22] Shaltynov, Askhat , Yulia Semenova , Madina Abenova , Assel Baibussinova , Ulzhan Jamedinova , and Ayan Myssayev . Apr 17 2024. “An Analysis of Financial Protection and Financing Incidence of Out‐Of‐Pocket Health Expenditures in Kazakhstan from 2018 to 2021.” Scientific Reports 14(1): 8869. 10.1038/s41598-024-59742-9. 38632372 PMC11024138

[23] Chu, Kathryn , Rebecca Maine , and Riaan Duvenage . Oct 2021. “We Asked the Experts: The Role of Rural Hospitals in Achieving Equitable Surgical Access in Low‐Resourced Settings.” World Journal of Surgery 45(10): 3016–3018. 10.1007/s00268-021-06271-5. 34338826 PMC8327595

[24] Mackintosh, Maureen , Amos Channon , Anup Karan , Sakthivel Selvaraj , Eleonora Cavagnero , and Hongwen Zhao . Aug 6 2016. “What Is the Private Sector? Understanding Private Provision in the Health Systems of Low‐Income and Middle‐Income Countries.” Lancet 388(10044): 596–605. 10.1016/S0140-6736(16)00342-1. 27358253

[25] Lockhart, Kathleen , Douglas Dunn , Shawn Teo , Jessica Y. Ng , Manvinder Dhillon , Edward Teo , and Mieke L. van Driel . Sep 13 2018. “Mesh versus Non‐mesh for Inguinal and Femoral Hernia Repair.” Cochrane Database of Systematic Reviews 9(9): CD011517. 10.1002/14651858.CD011517.pub2. 30209805 PMC6513260

[26] Beard, Jessica H. , Michael Ohene‐Yeboah , Stephen Tabiri , Joachim K. A. Amoako , Francis A. Abantanga , Carrie A. Sims , Pär Nordin , Andreas Wladis , Hobart W. Harris , and Jenny Löfgren . Sep 1 2019. “Outcomes after Inguinal Hernia Repair with Mesh Performed by Medical Doctors and Surgeons in Ghana.” JAMA Surg 154(9): 853–859. 10.1001/jamasurg.2019.1744. 31241736 PMC6596328

[27] Bentounsi, Zineb , Chris Lavy , Chiara Pittalis , Morgane Clarke , Jean Rizk , Grace Le , Ruairi Brugha , Eric Borgstein , and Jakub Gajewski . Feb 2021. “Which Surgical Operations Should Be Performed in District Hospitals in East, Central and Southern Africa? Results of a Survey of Regional Clinicians.” World Journal of Surgery 45(2): 369–377. 10.1007/s00268-020-05793-8. 33000309 PMC7773610

