# Supplementary material for: Provision of inguinal hernia surgery in first‐referral hospitals across low‐ and middle‐income countries: Secondary analysis of an international cohort study
Source: World J Surg. 2024 Nov 22;49(2):374–84. doi: 10.1002/wjs.12374 (PMC11798680; doi:10.1002/wjs.12374)
Supplement: Supplementary file 1 — Supplementary Material [file WJS-49-374-s001.docx]

**Supplement**

**Provision of inguinal hernia surgery in first referral hospitals across low- and middle-income countries: secondary analysis of an international cohort study**

NIHR Global Health Research Unit on Global Surgery

A full list of collaborating authors is shown in Appendix 1

**Joint corresponding authors:**

Dr Philip Alexander, Lady Willingdon Hospital, Manali, Kullu District, Himachal Pradesh, India

philalex1@gmail.com

&

Dr Maria Picciochi, NIHR Global Health Research Unit on Global Surgery, Institute of Applied Health Research, University of Birmingham, Birmingham B15 2TH, UK

m.picciochi@bham.ac.uk

**Funding:** This study was supported by NIHR Global Health Research Unit Grant (NIHR133364) and a project research grant from Portuguese Hernia and Abdominal Wall Society (Sociedade Portuguesa de Hernia e Parede Abdominal). The funders had no role in study design or writing of this report. The views expressed are those of the authors and not necessarily those of the National Health Service, the NIHR or the UK Department of Health and Social Care.

**Appendices**

| Section 1 – List of authors | Pages 1-20 |
| --- | --- |
| Section 2 – Supplementary tables and materials | Pages 21-28 |

**Section 1: List of authors**

**Writing group:** M Picciochi, PV Alexander, T Anyomih, N Boumas, R Crawford, F Enoch Gyamfi, N Hopane, M Isiagi, SK Kamarajah, V Ledda, A Matei, A Mulliez, D Nepogodiev, N Roy, CE Okereke, R Tubasiime, M Steinruecke, A Bhangu (senior author).

**Study Management Group:** M Picciochi, AO Ademuyiwa, A Adisa, AE Agbeko, JA Calvache, D Chaudhry, R Crawford, AC Dawson, M Elhadi, A Ghaffar, D Ghosh, J Glasbey, PD Haque, E Harrison, A Isik, I Jakaityte, SK Kamarajah, O Kouli, I Lawani, S Lawani, V Ledda, E Li, J Martin, A Minaya Bravo, D Morton, D Nepogodiev, F Ntirenganya, O Omar, SZY Ooi, R Oppong, F Pata, A Ramos-De la Medina, M Sampaio-Alves, JFF Simoes, M Steinruecke, S Tabiri, A Bhangu.

**Dissemination Committee** (listed by country)**:**

Albania: I Dajti; Algeria: Z Djama; Argentina: M Lucchini, RM Palacios Huatuco; Australia: K Atherton, AC Dawson, E Lun; Austria: F Aigner; Belgium: F Berrevoet; Benin: I Lawani, S Lawani, C Bokossa; Bosnia and Herzegovina: S Delibegovic; Bulgaria: M Slavchev; Burkina Faso: AF Sanon, A Sanou; Burundi: JB Gusa, JC Mbonicura; Cameroon: A Bang, O Gabom, C Nwegbu; Canada: A Brar, J Martin; Chile: MM Modolo, M Olivos; Colombia: JA Calvache; Croatia: J Mihanovic; Cyprus: N Gouvas, A Yiallourou; Czech Republic: B East; Dominican Republic: S Batista, R Rivas; Ecuador: EP Lincango; Egypt: S Emile; Ethiopia: AB Aregawi; France: AP Arnaud; Gabon: N Boumas; Georgia: Z Demetrashvili; Germany: H Lederhuber, MW Löffler; Ghana: AE Agbeko, NB Sam, S Tabiri, F Agyei, FE Gyamfi, S Mohammed; Greece: I Katsaros, G Tsoulfas; India: L Bains, J Dhiman, D Ghosh, PD Haque, A Suroy; Ireland: S Ramjit; Israel: G Marom; Italy: F Pata, G Gallo; Jordan: F Ayasra; Kazakhstan: I R Fakhradiyev; Kenya: IHS Hamdun; Kyrguzstan: A Iqbal; Liberia: E Mbanzabugabo; Libya: M Elhadi; Lithuania: A Gulla; Madagascar: L Samison; Malawi: M Nyirenda, R Nyirenda; Malaysia: AC Roslani; Mali: B Bengaly; Malta: J Psaila; Mexico: L Martinez, A Ramos-De la Medina; Namibia: PR Nashidengo; New Zealand: M McGuinnes, D Wright; Niger: A Ousseini; Nigeria: A Adisa, AO Ademuyiwa; North Macedonia: T Risteski; Oman: Z Al Balushi, B Dawud, A AlSharqi, F Ali; Pakistan: AU Qureshi; Palestine: H Abu-Arish; Paraguay: H Gomez-Fernandez; Philippines: JM Faylona, MD Sacdalan; Poland: W Krawczyk; Portugal: JG Goncalves-Nobre, M Sampaio-Alves, I Santos; Romania: I Negoi; Russian Federation: A Butyrskii; Rwanda: JC Allen, F Ntirenganya; Sierra Leone: I Fortune; Slovenia: J Kosir; South Africa: N Parker, K Chu; Spain: A Minaya Bravo; Sri Lanka: D Wickramasinghe, U Jayarajah; Sudan: M Elmujtaba; Sweden: M Nikberg; Switzerland: E Gialamas; Syria: M Alshaar; Tanzania: M Nkoronko; Turkey: A Isik; Uganda: I Mubesi; United States: J Ng-Kamstra; Venezuela: O Bahsas-Zaky.

**Data handling and management:** M Picciochi, R Acharya, D Badran, A Chaudhry, JG Goncalves-Nobre, RR Gujjuri, B Kadir (senior statistician), SR Knight, S Lawday, O Omar (senior statistician), KSY Ooi, R Ooi, C Varghese.

**Hospital leads** (listed by country and city)**:**

Albania: E Agastra (Korca; Regional Hospital of Korca); I Dajti (Tirana; University hospital Koco Gliozheni).

Algeria: R Belouz (Algiers; CHU Isaad Hassani); Z Djama (Constantine; University Hospital Abdelhamid Ben Badis); A Mouffokes (Oran; EHU-1st November 1954).

Argentina**:** ME Muriel (Allende, Cordoba; Sanatorio Allende - Sede Cerro); RM Palacios Huatuco (Buenos Aires; Hospital Italiano de Buenos Aires); M Santillan (Buenos Aires; Hospital Universitario CEMIC); A Duro (Buenos Aires; Hospital municipal de vicente lópez); JI Valenzuela (City of Buenos Aires; Hospital Velez Sarsfield); DA Pantoja Pachajoa (Cordoba; Clinica Universitaria Reina Fabiola); G Romero reyna (Cordoba; Sanatorio Allende - Sede Nueva Cordoba); CM Florián Villa (San Francisco; Clinica Regional del Este).

Bangladesh: S Islam (Dhaka; Dhaka Medical College Hospital); MP Singh (Dinajpur; Lamb Hospital).

Benin**:** G Gbessi (Cotonou; Centre National Hospitalier et Universitaire Hubert Koutoukou Maga); H Aouagbe Behanzin (Cotonou; Hopital de Menontin); M Agbadebo (Dassa-Zoumè; Hôpital de Zone de Dassa-Zoumè); E Bara (Kandi; Hôpital de zone de Kandi); AB Yevide (Klouékanme; Hopital de Zone de Klouékanme); TK Hessou (Natitingou; Centre Hospitalier Départemental de l’Atacora); AM Hodonou (Parakou; Centre Hospitalier Universitaire Borgou Alibori); I Lawani (Porto Novo; Centre Hospitalier Universitaire et Departemental Oueme Plateau).

Bosnia and Herzegovina: Z Matkovic (Doboj; Genera Hospital ‘Sveti aposto Luka’ Doboj); N Lalovic (Foča; University Hospital Foča); J Miskovic (Mostar; SKB University Clinical Hospital Mostar); M Salibašić (Sarajevo; Clinical Center University of Sarajevo); A Cerovac (Tešanj; General Hospital Tešanj); A Tursunovic (Tuzla; University Clinical Center Tuzla).

Brazil: C Panis (Francisco Beltrão; Universidade Estadual do Oeste do Paraná).

Bulgaria: T Ivanov (Pleven; Heart and Brain - Pleven Hospital); M Karamanliev (Pleven; University Hospital Dr Georgi Stranski, Medical University - Pleven); R Donchev (Plovdiv; MHAT St. Karidad); D Hadzhiev (Plovdiv; UMHAT Sveti Georgi); T Yotsov (Ruse; University Hospital Medika); E Hristova (Sofia; Fifth City Hospital Sofia - 5th MBAL).

Burkina Faso**:** AF Sanon (Ouagadougou; Tengandogo University Hospital).

Burundi**:** JC Mbonicura (Bujumbura; Centre Hospitalo-Universitaire de Kamenge); N Diomede (Bujumbura; Kamenge Military Hospital); J Gusa (Bujumbura; Prince Regent Charles Hospital).

Cambodia: S Stock (Battambang; Handa Medical Centre).

Cameroon: O Ndizeye (Bamenda; Nkwen Baptist Hospital).

China: W Yang (Guangzhou; The First Affiliated Hospital of Jinan University).

Colombia: CJ Perez Rivera (Bogota; Fundacion Cardioinfantil-IC); S Sierra (Medellin; Clínica CES); DS Garcés Palacios (Popayan; Hospital Susana Lopez de Valencia); JA Calvache (Popayán; Hospital Universitario San José).

Dominican Republic: R Rivas (Santo Domingo; CEDIMAT - Centro de Diagnóstico, Medicina Avanzada, Laboratorio y Telemedicina).

Egypt: G Abouelnagah (Alexandria; Alexandria Main University Hospital); D Attia (Alexandria; Alexandria Medical Research Institute); G Abouelnagah (Alexandria; Smouha University Hospital); A Maher (Assiut; Assiut University Children Hospital); A Kedwany (Assiut; Assiut University Hospital); S Abdelmohsen (Aswan; Aswan University Hospital, Aswan University); R Adel Diab (Cairo; Al Zahraa University Hospital); A Al-Mallah (Cairo; Al-Azhar University Hospitals); H Taher (Cairo; Cairo University Children’s Hospitals (CUSPH & CUCH)); H Abozied (Cairo; EL-Hussein University Hospital, Al-Azhar University, Faculty Of Medicine); ASM Abdelrahman (Cairo; Giza International Hospital); M ElFiky, N Shehata (Cairo; Kasr Al Ainy Faculty of Medicine, Cairo University); B Fahmy (Giza; The Memorial Soaad Kafafi University Hospital); H Elghadban (Mansoura; Mansoura University Hospital); E Adel Mahmod Sultan (Menofia; Menofia University Hospital); M Omar (Qena; Qena University Hospital); EA Ahmed (Sohag; Sohag University Hospital); AM Elkhouly (Tanta; Tanta University Hospital); A Asla (Zagazig; Al Ahrar Zagazig Teaching Hospital).

Ethiopia: F Terefe (Addis Ababa; Yekatit 12 hospital medical college); A Yeshitila (Deberebirhan; Hakim Gizaw Hospital); M Taeme (Dessie; Dessie Referral Hospital); A Demessie (Gondar; Gondar University Comprehensive specialized hospital); AB Aregawi (Hawassa; Hawassa University Comprehensive Specialized Hospital); N S.Bayleyegn (Jimma; Jimma University Medical Center); B Sime (Yirgalem; Yirgalem Hospital Medical College).

Gabon: N Boumas (Libreville; Centre Hospitalier universitaire mère enfant Fondation Jeanne Ebori).

Georgia: Z Demetrashvili (Tbilisi; N.Kipshidze Central University Clinic).

Ghana: R Armah (Accra; Greater Accra Regional Hospital); A Bediako Bowan (Accra; Korle-Bu Teaching Hospital); E Kafui Ayodeji (Accra; Pentecost Hospital); EA Arkoh (Ankaful; Ankaful Leprosy General Hospital); FE Gyamfi (Berekum; Berekum Holy Family Hospital); A Davor (Bolgatanga; Upper East Regional Hospital); MT Morna (Cape-Coast; Cape Coast Teaching Hospital); N Agboadoh (Damongo; St. Anne’s Hospital); EA Nachelleh (Ho; Ho Teaching Hospital); AE Agbeko (Kumasi; Komfo-Anokye Teaching Hospital); S Mensah (Kumasi; University Hospital, KNUST); P Taah-Amoako (Nsawkaw; Tain District Hospital); K Collins (Sunyani; Brong-Ahafo Regional Hospital); AS Seidu (Tamale; Tamale Teaching Hospital); BK Seshie (Tema; Tema General Hospital); G Ansong (Walewale; Walewale Government Hospital).

Guatemala: M Aguilera-Arevalo (Guatemala City; Hospital General San Juan De Dios); L Talé-Rosales (Guatemala City; Hospital Juan Jose Arevalo Bermejo); ST Torres Rodríguez (Guatemala City; Hospital de Referencia Nacional de Enfermedades Respiratorias).

India: N Krishnappa (Bangalore, Karnataka; BGS Global Institute of Medical Sciences); S Kumar Venkatappa (Bangalore; Victoria Hospital); TS Mishra (Bhubaneswar; All India Institute Of Medical Sciences - Bhubaneswar); Y Sakaray (Chandigarh; Postgraduate Institute of Medical Education & Research, Chandigarh, India); R Kottayasamy Seenivasagam (Coimbatore; PSG Institute of Medical Sciences and Research); R Sharma (DELHI; St Stephen’s Hospital); R Gupta (Dehradun; Synergy Institute of Medical Sciences); M Luthra (Delhi; Holy Family Hospital); T Longkumer (Dimapur; Christian Institute of Health Sciences and Research); A Chhabra (Faridkot; Guru Gobind Singh Medical College & Hospital (Baba Farid University of Health Sciences)); T Doma Bhutia (Gangtok; Sir Thutob Namgyal Memorial Hospital Sochakgang); M Pathak (Jodhpur; All India Institute of Medical Sciences (AIIMS), Jodhpur); J Rathod (Karamsad; Shree Krishna Hospital); MK Agrawal (Lucknow; King George’s Medical University); D Jain (Ludhiana; Christian Medical College & Hospital); NK Chaudhry (Ludhiana; Satguru Partap Singh Hospital); A Mathew (Madhepura; Madhepura Christian Hospital); P Alexander (Manali; Lady Willingdon Hospital); V Kumar (Manipal; Kasturba Medical College Hospital, Manipal); RD Sharma (Mumbai; Lilavati Hospital & Research Centre); B Sarang (Mumbai; Terna Medical College and Hospital); D Singh (Nandurbar; Chinchpada Christian Hospital); N Gupta (New Delhi; ABVIMS Dr RML Hospital); S Kulkarni (New Delhi; Army Hospital Research & Referral New Delhi); T Rashid (New Delhi; Hamdard Institute of Medical Sciences & Research); L Bains (New Delhi; Maulana Azad Medical College); T Iahmo (Padhar; Padhar Hospital); A Kumar (Patiala; Government Medical College Patiala); M Kumar (Patna; All India Institute of Medical Sciences, Patna); R Abhinaya (Pondicherry; Jawaharlal Institute of Postgraduate Medical Education and Research); VS Jha (Pune; Command Hospital, Southern Command); D Dugar (Raipur; All India Institute of Medical Sciences Raipur); S Basu (Rishikesh; All India Institute Of Medical Sciences); K Singh (SAS Nagar (Mohali) ; BR Ambedkar State Institute of Medical Sciences Mohali); C Mahakalkar (Sawangi (Meghe), Wardha; Acharya Vinoba Bhave Rural Hospital); FQ Parray (Srinagar; Sher-i-Kashmir Institute of Medical Sciences); JA Kalyanapu (Tezpur; Baptist Christian Hospital); M Chisthi (Thiruvananthapuram; Government Medical College Thiruvananthapuram); A Kavalakat (Thrissur; Jubilee Mission Medical College & Research Institute); B Roopavathana. S (Vellore; Christian Medical College & Hospital).

Iran, Islamic Rep.: N Yousefzadeh Kandevani (Bastak; Farabi hospital); M Pourfridoni (Jiroft; Imam Khomeini Hospital).

Iraq: R Raheem Attallah Al_obaidy (Anbar; Heet General hospital); Z Alkhuzaie (Najaf; Al Batool private hospital); MA Al-Juaifari (Najaf; Al-Najaf Al-Ashraf Teaching Hospital).

Jordan: S Alananzeh (Ajloun; Al Iman Hospital); A Qasem (Amman; Al-Basheer Hospital); Y Alawneh (Amman; Ibn Al Haitham Hospital); S Al-Tahayneh (Amman; Islamic Hospital); A Khamees (Amman; Jordan University Hospital); MEH Albanna (Amman; Marka Specialty Hospital); RKZ Almahadin (Amman; Prince Hamza hospital); M Mahafdah (Ar Ramtha; King Abdullah University Hospital/ Jordan University of Science and Technology); O Mansour (As-Salt; Al Hussain New Salt Hospital); M Mubarak (Irbid; Ar Ramtha Govermental Hospital); A Alrababah (Irbid; Princess Basma Hospital).

Kazakhstan: M Kulimbet (Almaty; City Clinical Hospital No.7, Asfendiyarov Kazakh National Medical University); I Fakhradiyev (Almaty; JSC ‘Central Clinical Hospital’, Asfendiyarov Kazakh National Medical University).

Kenya: R Parker (Bomet; Tenwek Hospital).

Lebanon: D Rahme (Beirut; Hopital Libanais Geitaoui); H Hamdar (Jbail ; Maritime Hospital).

Libya: W Ebrahim (Albayda; Albayda Medical Center); M Saleh (Benghazi; Al-jalaa Teaching/Trauma Hospital); S Alsaeiti (Benghazi; Benghazi Children’s Hospital); R Michael (Benghazi; Benghazi Medical Center); A Bojazyah (Darna; Al-Wahda Hospital); H Bileid Bakeer (Gharyan; Gharyan Central Hospital); M Abudabbous, N Albahloul (Misurata; Misurata Central Hospital); A Egdeer (Nalut; Nalut Central Hospital); H Embarek (Sebha; Al-Majd Clinic); M Abdelkabir (Sebha; Aseel Alghad Clinic); H Idheiraj (Sebha; Sabha Medical Center); R Salim (Tobruk; Tobruk Medical Center); K Ayad (Tripoli; Alkhalil hospital); A Alragheai (Tripoli; Metiga Hospital); S Egreara (Tripoli; Sabratha teaching hospital); S Timmalah (Tripoli; Tripoli Medical Center/ Tripoli University Hospital); N Lahmer (Zawia; Zawia Teaching Hospital); N Ben Hasan (Zliten; Zliten Teaching Hospital).

Madagascar: F Rasoaherinomenjanahary (Antananarivo; Joseph Ravoahangy Andrianavalona Hospital).

Malaysia: MS Mohd Shah (Kelantan; Hospital Universiti Sains Malaysia); R Noor (Kota Bharu; Hospital Raja Perempuan Zainab II); SN Loke (Kuching, Sarawak; Sarawak General Hospital); MA Yunus (Malacca; Hospital Jasin); H Amin-Tai (Serdang; Hospital Pengajar Universiti Putra Malaysia (HPUPM)).

Mali: KS Dembele (Ségou; District Hospital of Tominian).

Mexico: CM Nuño-Guzmán (Guadalajara; Antiguo Hospital Civil de Guadalajara); LA Flores Chávez (Guadalajara; Clínica de Especialidades más Centro de Cirugía Simplificada); CM Nuño-Guzmán, G Yanowsky-Reyes (Guadalajara; Hospital Civil Fray Antonio Alcalde); A Gonzalez Ojeda (Guadalajara; Hospital de Especialidades, CMNO-IMSS); G Ambriz González (Guadalajara; UMAE Hospital de Pediatria Centro Medico Nacional de Occidentes IMSS); EE Lozada Hernandez (León; Hospital Regional e Alta Especialidad del Bajio); M Trejo-Avila (Mexico City; Hospital General Dr. Manuel Gea González); C Moreno-Licea (Mexico City; Instituto Nacional de Ciencias Médicas y Nutrición ‘Salvador Zubirán’); A Navarrete-Peón (Pachuca; Sociedad Española de Beneficencia); M Noguez Castillo (Querétaro; Hospital de especialidades del niño y la mujer); A Ramos-De la Medina (Veracruz; Hospital Español Veracruz).

Morocco: I Gouazar (Marrakech; Centre Hospitalier Universitaire Mohammed VI, Marrakech); A Ouachhou (Rabat; Centre Hospitalier Universitaire Ibn Sina Rabat).

Namibia: PR Nashidengo (Windhoek; Windhoek Central Academic Hospital).

Nigeria: A Adeyeye (Ado Ekiti; Afe Babalola University Multi-System Hospital); J Olaogun (Ado-Ekiti; Ekiti State University Teaching Hospital); N Oloko (Bauchi; Abubakar Tafawa Balewa University Teaching Hospital Bauchi); P Agbonrofo (Benin City; University of Benin Teaching Hospital); A Abiodun (Bida; Federal Medical Centre Bida); U Ezomike (Enugu; University of Nigeria Teaching Hospital); SA Sani (Gwagwalada; University of Abuja Teaching Hospital); TA Lawal (Ibadan; University College Hospital); AO Lawal (Idi Araba; Lagos University Teaching Hospital); AI Okunlola (Ido Ekiti; Federal Teaching Hospital, Ido Ekiti); OM Williams (Ikeja; Lagos State University Teaching Hospital); A Adisa (Ile-Ife; Obafemi Awolowo University Teaching Hospitals Complex); T Mohammed (Ilesa; Obafemi Awolowo University Teaching Hospitals Complex Wesley Guild Hospital Unit); P Elemile (Ilishan-Remo; Babcock University Teaching Hospital); II Aremu (Ilorin; General Hospital); L Abdur-Rahman (Ilorin; University of Ilorin Teaching Hospital); JG Makama (Kaduna; Barau Dikko Teaching Hospital); IU Garzali (Kano; Aminu Kano Teaching Hospital); TT Ibiyeye (Lokoja; Federal Teaching Hospital Lokoja); OH Ekwunife (Nnewi; Nnamdi Azikiwe University Teaching Hospital); OH Ekwunife (Onitsha; Holy Rosary Specialist Hospital); O Ojewuyi (Osogbo; UNIOSUN Teaching Hospital); I Ogundele (Sagamu; Olabisi Onabanjo University Teaching Hospital); M Daniyan (Zaria; Ahmadu Bello University Teaching Hospital).

North Macedonia: T Risteski (Skopje; University Clinic for Pediatric Surgery).

Pakistan: S Ahmed (Islamabad; Dr Akbar Niazi Teaching Hospital); SH Waqar (Islamabad; The Pakistan Institute of Medical Sciences); M Shahid (Karachi; PAF Faisal Hospital); F Ashraf (Karachi; Patel Hospital); AN Syed (Karachi; The Indus Hospital); AS Ammar (Lahore; Bahria International Hospital, Bahria Orchard); K Hayat (Lahore; Services Hospital Lahore); N Talat (Lahore; The Children’s Hospital & The Institute of Child Health Lahore); W Mabood (Peshawar; Mercy Teaching Hospital); HW Bhatti (Rawalpindi; Benazir Bhutto Hospital); M Usman Malik (Sargodha; District Headquarter & Teaching Hospital - Sargodha).

Palestine: B Mohamad (Bethlehem, West Bank; Beit Jala Governmental Hospital (Al Hussein)); A Alwali (Gaza; Al-Shifa Hospital); A AbuNemer (Gaza; Nasser Hospital); S Alijla (Gaza; Palestine Red Crescent Society - Al-amal Hospital); H Ayesh (Hebron, West Bank; Al-Ahli Hospital); H Abu-Arish (Hebron, West Bank; Governmental Hebron Hospital-Alia); D Rabaia, A Jaber (Jenin, West Bank; The Martyr Dr. Khalil Sulaiman Hospital (Jenin Governmental Hospital)); M MohammedAli (Nablus, West Bank; Rafidia Hospital); A Attili (Tulkarm, West Bank; Martyr Thabet Thabet Govermental Hospital).

Paraguay: OM Cuenca Torres (Asuncion; Hospital de Clínicas, II Cátedra de Clínica Quirúrgica, Universidad Nacional de Asunción).

Peru: V Panduro-Correa (Huánuco; Hospital Regional Hermilio Valdizán Medrano); L Fuentes Rivera Lau (Lima; British American Hospital); CF Huaroto Landeo (Lima; Clinica Internacional); G Mendiola (Lima; Hospital Santa Rosa de Lima); Y Carpio Colmenares (Lima; SANNA - Clínica El Golf); C Shiraishi Zapata (Paita; Hospital I Miguel Cruzado Vera EsSalud); R Díaz-Ruiz (Piura; Jose Cayetano Heredia III Regional Hospital).

Russian Federation: V Kakotkin (Kaliningrad; Immanuel Kant Baltic Federal University, Regional Clinical Hospital); A Bedzhanyan (Moscow; Petrovsky National Research Centre of Surgery); S Katorkin (Samara; Hospital Surgery Clinic of Samara State Medical University); A Butyrskii (Simferopol; Municipal Emegency Hospital No.6); V Ten (Yuzhno-Sakhalinsk; Private healthcare institution ‘RZD-Medicine’).

Rwanda: N Christian (Huye, Gisagara; Butare university teaching hospital (CHUB)); C Mpirimbanyi (Kigali; Kibagabaga Hospital); A Costas-Chavarri (Kigali; Rwanda Military Hospital).

Serbia: A Karamarkovic (Belgrade; Zvezdara University Medical Center).

South Africa: M Flint (Cape Town; Groote Schuur Hospital); A Victor (Cape Town; Karl Bremer Hospital); SS Verhage (Cape Town; Khayelitsha District Hospital); F Gool (Cape Town; Mitchell’s Plain District Hospital); T Mabogoane (Cape Town; Victoria Hospital Wynberg); B Phakathi (Durban; King Edward VIII Hospital); V Pillay (Durban; Stanger Hospital); R Jayakrishnan (East London; Cecilia Makiwane Hospital); Y Manickchund (East London; Frere Hospital); O Jolayemi (Empangeni; Ngwelezana Hospital); H Stark (George; George Hospital); C Molewa (Johannesburg; Edenvale); H Wain (Pietermaritzburg; Edendale Hospital); D Montwedi (Pretoria; Kalafong Academic Hospital); G De Wee (Upington; Dr Harry Surtie Hospital); C Dempers (Worcester; Worcester Provincial Hospital).

Sri Lanka: S Srishankar (Anuradhapura; Teaching Hospital Anuradhapura); D Wickramasinghe (Colombo; National Hospital of Sri Lanka); U Jayarajah (Dehiwala; Colombo South Teaching Hospital); S Gobishangar (Jaffna; Teaching Hospital, Jaffna); W Wijenayake (Werahera; University Hospital, Kotelawala Defence University).

Sudan: I Abdalla (Ed Dueim; Ed Dueim Teaching Hospital); S Ibrahim Tour Harakan (El Geneina; El Geneina teaching Hospital); MM Yassin (Gadarif city; Gadarif teaching hospital); Z Aljalabi (Kassala; Police hospital); I Adel (Khartoum; Bashair Teaching Hospital); IMG Ahmed (Khartoum; Ibrahim Malik Teaching Hospital); M Hajalamin (Khartoum; Omdurman Teaching Hospital); EE Abuobaida Banaga Hag El Tayeb (Khartoum; Ribat university hospital); HA Fadlalmola (Khartoum; Soba University Hospital); E Alkhalifa (Wad Madani; University of Gezira Hospital).

Syrian Arab Republic: MA Farho (Aleppo; Abd Al Wahab Agha Hospital); AA Kayali (Aleppo; Al-Shahbaa Private Hospital); M Aloulou (Aleppo; Aleppo Private Hospital); A Ghazal (Aleppo; Aleppo University Hospital); B Alsaid (Damascus; Al assad university hospital); M Klib (Damascus; Al-Mouwasat University Hospital); H Dalati (Damascus; Children’s University Hospital); S Jomaa (Damascus; Damascus Hospital); Y Alhammoud (Homs; Al-Basel Specialized Hospital in Karm El-Louz); S Abbas (Homs; The Military Hospital); G Hneino (Latakia; Al Saydeh Surgical Hospital); I Ali (Latakia; National Hospital); G Bashour (Latakia; Othaman Hospital); A Hammed (Latakia; Tishreen University Hospital).

Thailand: S Techapongsatorn (Bangkok; Vajira hospital).

Togo: F Alassani (Lomé; CHU Sylvanus Olympio).

Tunisia: A Sebai (Tunis; La Rabta Hospital).

Turkey: GC Bulbuloglu (Adana; Adana Seyhan State Hospital); MA Koç (Ankara; Ankara University Medical School); MY Uzunoglu (Bursa; Bursa City Hospital); B Yigit (Elazig; Elazig Fethi Sekin City Hospital); AN Sanli (Gaziantep; Abdulkadir Yuksel State Hospital); GK Kurtoglu (Istanbul; Acibadem Altunizade Hospital); E Tuzuner (Istanbul; Acibadem Maslak Hospital); Y Altinel (Istanbul; Bagcilar Research And Training Hospital); ÖP Zanbak Mutlu (Istanbul; Bahçelievler State Hospital); RE Sönmez (Istanbul; Istanbul Medeniyet University, School of Medicine); S Bektas (Istanbul; Istanbul Medipol University Hospital); E Erginöz (Istanbul; Istanbul universty - Cerrahpaşa Medical faculty); A Özcan (Istanbul; Kanuni Sultan Suleyman Training and Research Hospital); Y Tosun (Istanbul; Kartal Dr. Lutfi Kirdar Training and Research Hospital); İH Özata (Istanbul; Koç University Medical School); TK Uprak (Istanbul; Marmara University, School of Medicine); E Unal (Istanbul; Sehit Prof.Dr. İlhan Varank Training and Research Hospital); N Kiziltoprak (Istanbul; Sultan 2. Abdulhamid Han Training and Research Hospital, University of Health Sciences); M Ergenç (Istanbul; Sultanbeyli State Hospital); MT Demirpolat (Istanbul; University of Health Science Umraniye Education and Research Hospital); B Citgez (Istanbul; Uskudar University Faculty of Medicine, Memorial Hospital); H Ulman (Izmir; Bakircay University Cigli Training and Research Hospital); YK Şen (Izmir; University of Health Sciences Izmir Bozyaka Training and Research Hospital); E Colak (Samsun; Samsun University Samsun Training and Research Hospital); N Kavak (Zonguldak; Zonguldak Bulent Ecevit University School of Medicine Research and Training Hospital); E Kamer (İzmir; University of Health Sciences Tepecik Training and Research Hospital).

Uganda: I Mubezi (Iganga; Iganga district hospital); H Lule (Kigumba; Kiryandongo Hospital); S Stonelake (Luwero; Kiwoko Hospital).

Yemen, Rep.: MY Abdualqader (Hajjah; Kowaydina hospital); B Alshaikh (Sana’a; Al-Thawra Modern General Hospital).

**Collaborators** (listed by country and city)**:**

Albania: B Ibi, S Faber (Korca; Regional Hospital of Korca); I Dajti (Tirana; University hospital Koco Gliozheni).

Algeria: K Bensmain, ZR Benamrouche , IE Boumakhlouf, IE Boudis, H Abdoun , M Benamrouche , M Saidani (Algiers; CHU Isaad Hassani); Z Djama, A Chied, HA Mimouni (Constantine; university hospital abdelhamid ben badis); AK Awad, B Radja, B Abdennour , MN Bouhafs, MEA Meghaizerou (Oran; EHU-1st November 1954).

Argentina: P Carmignani, J Mondino, R Figueroa, J Morales, FR Pascual (Allende, Cordoba; Sanatorio Allende - Sede Cerro); MA Bequis, F Suldrup, C Korzin, J Napoli , N Feijoo, F Mahnic, ME Duran, L Chantada, C Brandi, JF Viñas, F Lucero, C Samojeden, LJ Caram, S Bertone, F Corvatta (Buenos Aires; Hospital Italiano de Buenos Aires); L Garciandia (Buenos Aires; Hospital Universitario CEMIC); M Rius, S Matthiess, J Paredes, MC Kalaydjian , A Veira (Buenos Aires; Hospital municipal de vicente lópez); MA Fernández Zurita, JI Valenzuela (City of Buenos Aires; Hospital Velez Sarsfield); S Gomez, GR Viscido, MA Doniquian (Cordoba; Clinica Universitaria Reina Fabiola); R Badra, JS García, CI Ferrero, M Garcia, L Granero (Cordoba; Sanatorio Allende - Sede Nueva Cordoba); M Pagani (San Francisco; Clinica Regional del Este).

Bangladesh: K Nahar, T Akter (Dhaka; Dhaka Medical College Hospital); A Oosterkamp (Dinajpur; Lamb Hospital).

Benin: G Gbessi, J Avakoudjo, M Fiogbe, P Assouto, SP Chigblo (Cotonou; Centre National Hospitalier et Universitaire Hubert Koutoukou Maga); H Aouagbe Behanzin , M Seto, G Mevognon (Cotonou; Hopital de Menontin); M Agbadebo, A Hada, SFA Houndji (Dassa-Zoumè; Hôpital de Zone de Dassa-Zoumè); E Bara (Kandi; Hôpital de zone de Kandi); AB Yevide, ZF Tamou, E Hatangimana, B Cakpo , R Soglonou (Klouékanme; Hopital de Zone de Klouékanme); TK Hessou, SR Tobome, M Zounon (Natitingou; Centre Hospitalier Départemental de l’Atacora); AM Hodonou, C Bokossa , F Hounde , R Alinde (Parakou; Centre Hospitalier Universitaire Borgou Alibori); F Dossou, R Goudou, ACS Toi , G Natchagande (Porto Novo; Centre Hospitalier Universitaire et Departemental Oueme Plateau).

Bosnia and Herzegovina: M Stjepanovic (Doboj; Genera Hospital ‘Sveti aposto Luka’ Doboj); O Čančar, M Pejović (Foča; University Hospital Foča); J Miskovic, M Boras, M Kajic, V Dragisic, Z Brekalo, I Mikulic, N Soldo, M Bevanda, M Faletar (Mostar; SKB University Clinical Hospital Mostar); M Salibašić, E Hodžić, E Halilović, M Kruščica, E Bičakčić (Sarajevo; Clinical Center University of Sarajevo); A Cerovac, H Škiljo, E Hodžić, O Bedak, M Kalabić, E Begunić (Tešanj; General Hospital Tešanj); A Huremovic, E Alić (Tuzla; University Clinical Center Tuzla).

Brazil: RA Tenfen Carneiro (Francisco Beltrão; Universidade Estadual do Oeste do Paraná).

Bulgaria: D Georgiev, I Fidoshev, V Neykov, E Daleva, I Ilieva (Pleven; Heart and Brain - Pleven Hospital); M Karamanliev, D Dimitrov, A Shanker, P Vladova, MD Shoshkova, A Mehta, M Abdullahi, V Ratheesh, V Kamalathevan, C Wiesner, S Shittu, M Galasyuk, S Shanker (Pleven; University Hospital Dr Georgi Stranski, Medical University - Pleven); M Imirski, A Soumpasis (Plovdiv; MHAT St. Karidad); E Hadzhieva, D Chakarov (Plovdiv; UMHAT Sveti Georgi); T Yotsov, P Kamenova, A Vricheva, I Yotsov (Ruse; University Hospital Medika); E Hristova, K Spassov (Sofia; Fifth City Hospital Sofia - 5th MBAL).

Burkina Faso: A Sanou, M Windsouri, R Doamba, IW Bahikoro , AST Sanon (Ouagadougou; Tengandogo University Hospital).

Burundi: N Ildephonse, N Steve , Y Fulgence , GD Nibogora, C Nimbona , B Paul (Bujumbura; Centre Hospitalo-Universitaire de Kamenge); G Kazobinka, C Rukundo, N Renovat, E Ndizeye , M Dauphin (Bujumbura; Kamenge Military Hospital); FF Irakiza, L Niyidukunda, G Nkunguzi, N Oscar, N Theophile, B Révérien (Bujumbura; Prince Regent Charles Hospital).

Cambodia: S Oum, S Eam (Battambang; Handa Medical Centre).

Cameroon: NS Bibila, NN Cabrel, J Dongmo (Bamenda; Nkwen Baptist Hospital).

China: J Wang (Guangzhou; The First Affiliated Hospital of Jinan University).

Colombia: L García-Zambrano, PA Cabrera Rivera, N Paez, SV Agudelo Mendoza, MS Mosquera Paz, A Kadamani Abiyomaa, CF Roman Ortega, F Casas J, B Guerra (Bogota; Fundacion Cardioinfantil-IC); JD Molina Marin, C Maya, C Vasquez Maya, B Dieck, F Zapata (Medellin; Clínica CES); VA Ruiz López, MA Ñañez (Popayan; Hospital Susana Lopez de Valencia); DC Cardona Gomez, A Rojas, DC Patiño García , LI Bolaños, C Pastás, DA Pérez Muñoz (Popayán; Hospital Universitario San José).

Dominican Republic: R Rivas, B Calcaño, J Michel, Y Perez, R Ubiñas, P Garcia-Dubus, S Batista, S Strachan (Santo Domingo; CEDIMAT - Centro de Diagnóstico, Medicina Avanzada, Laboratorio y Telemedicina).

Egypt: Y Tanas, Y Kerolous, Y El Okazy, M Mokhtar, M Lotfy, M AL Sayed, H Altabbaa, AGMM Abouelnagah, O Al Shaqran, D M. Awad, A Sabry, G Nagy, E Amer, M Khalil, A El Shamarka, B Sharaf eldin, AAA Aboshosha, A Farrag, H Sherif Farouk Ahmed Hassan, Y Badr (Alexandria; Alexandria Main University Hospital); Y Orabi, M Kamal matter, A Alrifaee (Alexandria; Alexandria Medical Research Institute); M Elnour , M Zahran, A Aladl, M Bahnacy, Y Seada, M Kotb, A Ragab, Y Farag, L Khalifa, M Elmiesiry , D Abdalaziz, I Maharem, O AbouHiekal, O Hany, S Hanna, Y Dean, A Faisal (Alexandria; Smouha University Hospital); M Mostafa, I Ali, T Sabra, H Ibrahim , A K. Ali, M Osman , A Eltayeb (Assiut; Assiut University Children Hospital); A Morad, MO Herdan , A Abdelshafi, M M. Nathan, M Shalkamy, M Hamada Takrouney, R Sayad, FA Monib, A A Elhars, MM Saad, A Rashad Temerik, AM Abbas, O Mohamed Mokbel, E AbdElBaset, A Barakat, Z Bady, S Arafa, Z Osama, A Elzanaty , S Salama (Assiut; Assiut University Hospital); MEM Madany (Aswan; Aswan University Hospital, Aswan University); A Khaity , R Adel Diab, A Ghazal, A Ehab, A Abd Elsattar (Cairo; Al Zahraa University Hospital); A El-bastwesy (Cairo; Al-Azhar University Hospitals); A Eisa, M Elesseily, R Radwan, Y Asar, D Waleed, S Tawfik , AF Nixon Fulli , MJI Albert, A Autiak Ayii Chol, AR AbdelHalim, B Azhar, H Al-derume , M Alqadasi, N Alasbahi (Cairo; Cairo University Children’s Hospitals (CUSPH & CUCH)); H Abozied, Y Ashour, Y Mohamed , M Abdelmaboud, H Abdelazim, AES El kady, M Omar, A Haty, M Abd Al-Fattah, I Tagreda , IM Kereet, AG Montaser, M Faisal, M Masoud , M ElSayed Metwally (Cairo; EL-Hussein University Hospital, Al-Azhar University, Faculty Of Medicine); ASM Abdelrahman, S Mansour (Cairo; Giza International Hospital); A Nabil, MMA Marei, A Elmosalamy, L ElGebaly, AM Allam, T Awad, H Taher, K Fayed, M Abdelfattah, DH Khattab, N Ali, A Saleh, K Nassim, NK Aly, I Abo Elhagag, M Doss, M Elzayat, Y Samer Morsy, M ElFiky (Cairo; Kasr Al Ainy Faculty of Medicine, Cairo University); M Erfan, M Zaazou , M Reda, M Kouta, M Mohamad Amin, I Guirguis, H Amir , S Aboseif, M Abdelhafez, O Agha, A Khairy , A Dawoud, M Hamoud Almahly, S Mostafa Yassin , A El-Sherbiney, A Adel, H Foda, D Ahmed, AS Elkhodary , AA Mansour (Giza; The Memorial Soaad Kafafi University Hospital); A Elghrieb, M Natey, A Elshazli Mahmoud , A Khalleefah, H Elfeki, M Shalaby, M Sadek, M Abdelmaksoud, M Mostafa, M Waseem, A Adel, A Azam, A Sakr, A Sanad, م عبدالفتاح (Mansoura; Mansoura University Hospital); H Foad, S Elnoamany, S Selim, DS Alrokh, A Hassanin (Menofia; Menofia University Hospital); M Alansary (Qena; Qena University Hospital); A Ragheb, M Fahmy , M Mehanny (Sohag; Sohag University Hospital); AGE Aboelnasr, KMG Mohammed, M Eissa, S Allam, M Kamar (Tanta; Tanta University Hospital); A Asla (Zagazig; Al Ahrar Zagazig Teaching Hospital).

Ethiopia: F Terefe, WA Zerefa, E Gallo , A Yingess, T Kebede, M Mesfin, G Alemayehu , SM Djote, T Girma, DA Muhie (Addis Ababa; Yekatit 12 hospital medical college); E Yeshialem, G Seyfu, F Tsige, A Yeshitila, N Solomon (Deberebirhan; Hakim Gizaw Hospital); M Worku, S Lakew, Y Melkamu, ST Workineh, M Beletachew (Dessie; Dessie Referral Hospital); B Mengesha, G Getachew , I Tesfahun, M Teressa , BG Chiman, AB Aregawi (Hawassa; Hawassa University Comprehensive Specialized Hospital); N S.Bayleyegn, YY Metaferia, TG Moges, D Mengiste, A Teshome Sahilemariam (Jimma; Jimma University Medical Center); B Sime, T Jemal (Yirgalem; Yirgalem Hospital Medical College).

Gabon: PC Nze Obiang (Libreville; Centre Hospitalier universitaire mère enfant Fondation Jeanne Ebori).

Georgia: Z Demetrashvili, G Devidze, G Pisarevi, L Petashvili, E Ekaladze, N Lekiashvili, I Pipia, A Tvaladze, G Kenchadze, K Khutsishvili (Tbilisi; N.Kipshidze Central University Clinic).

Ghana: R Armah, NA Christian, D Daary, S Akuffo, A Twumasi, AD Andani , J Oppong, E Agbowada, J Daleku, J Ampadu, W Afedo, Z Robertson, A Obbeng, DN Lee, D Ofosuhene (Accra; Greater Accra Regional Hospital); GD Brown, F Osman, FJ Eshun, C Banka, I Amankwaa, E Ametefe , G Owusu, J Nyamekye - Baidoo , O Okrah, G Birikorang, P Kumassah, J Dei-Asamoa, J Annan, C Akli-Nartey , D Alifoe , S Tsatsu, C Ansah Larbi, U Una, K Yalley, A Bediako Bowan (Accra; Korle-Bu Teaching Hospital); KB Oduro-Boateng , S Anim , H Adjei, S Dognia , A Oppong , C Markin, JL Ahale, NKA Obuobi (Accra; Pentecost Hospital); S Agana , EB Akakpo, F Galley (Ankaful; Ankaful Leprosy General Hospital); FE Gyamfi, S Segnitome , S Agordjor , D Adjei, D Kyeremeh, Y Sarpong, F Opoku Twene, CK Ntow-Boahen, SAA Atupra , V Siepaal , IN Bakaweri, AJ Tabiim , R Agyei Boakye, A Asare Twumasi (Berekum; Berekum Holy Family Hospital); R Akankoatuesi Apatewen, U Kanyan Kassim , B Owusu Ansah, F Amoako (Bolgatanga; Upper East Regional Hospital); V Kudoh, K Boakye-Acheampong , B Boakye, R Kpangkpari, MT Morna, GA Rahman , EO Ofori, L Adagrah Aniakwo, M Amoako-Boateng , D T. Enti, S Debrah, M Nortey, P Koggoh , P Mensah, MM Agyapong, T Agyen, V Etwire, Y Adofo-Asamoah , S Yussif, M Yigah (Cape-Coast; Cape Coast Teaching Hospital); B Maanikuu, F Kuubetersob B. N, D Powell, A Gbeadese , F Tierenye (Damongo; St. Anne’s Hospital); EA Nachelleh, DYD Agbley , N Jiagge , R Akpaka, D Labadah, N Naabo, R Guzmán Lambert, FJ Eshun, P Ntem, E Setsoafia, N Affram, BY Hernandez Cervantes, DB Osei, K Ewool, F Nyarko, MA Ali, MA Oyortey, I Hagbevor, ME Ashong , JN Anyorigiya (Ho; Ho Teaching Hospital); J Yorke, A Lovi, EO Osei, PA Boateng, R Oppong-Amoah, K Agbedinu, C Dally, SG Brenu, F Galley, FM Agbemafoh, I Kyei, C Aboah, AY Appiah-Kubi, B Nimako, M Aikins, M Adinku , A Opoku-Agyapong, J Adjei, R Sagoe (Kumasi; Komfo-Anokye Teaching Hospital); A Gyedu, PK Boateng, S Mensah, E Frimpong-manso (Kumasi; University Hospital, KNUST); P Taah-Amoako, S Tabiri (Nsawkaw; Tain District Hospital); F Owusu, P Yeboah Owusu (Sunyani; Brong-Ahafo Regional Hospital); EMT Yenli, AS Seidu, M Dason , M Amadu, GA Adoro, M Kyereh, I Osman , J Quansah, C Doku, A Darkwa Boateng, AM Muntaka, MA Dokurugu, R Nesco, M Yahaya, EDF Konlan, A Issaka, RA Ramirez Calas , M Sheriff, M Dery, V Dassah (Tamale; Tamale Teaching Hospital); BK Seshie, F Caiquo, M Dum, L Ackam, K Yalley, FDA Agbodo, A Baiden Amissah, D Ashitey , LD Bray, J Ofori, M Ishak (Tema; Tema General Hospital); G Ansong (Walewale; Walewale Government Hospital).

Guatemala: M Aguilera-Arevalo, M Rodríguez-Ordoñez, DA Sosa Méndez, M Sebastián-Mendoza, TA Salazar-Lorenzana, R Herrera, DE Reyes Rodríguez, P Vásquez , S Morales , J Gomez , C García-Salas, JR Asturias Luna, J Tabora-Zepeda, S Vasquez, JR Hernández , JOM Herrera Batres , D Muñoz, E Ayala , O Coyoy-Gaitán (Guatemala City; Hospital General San Juan De Dios); JB Pellecer Cano, DA Palma Portillo, M Blanco (Guatemala City; Hospital Juan Jose Arevalo Bermejo); D Herrera, SA Villeda , O Lima Azurdia (Guatemala City; Hospital de Referencia Nacional de Enfermedades Respiratorias).

India: S Jp, M Bhat, A Raheja, I Shariff, H Anand , B R Budihal, S Kashyap, B Arya, Y M S, N Krishnappa (Bangalore, Karnataka; BGS Global Institute of Medical Sciences); M Manangi, P Anandan, S Shivashankar chikkanayakanahalli, S Kumar Venkatappa (Bangalore; Victoria Hospital); TS Mishra, P Kumar, M Gureh, MK Sethi, AA Asharaf (Bhubaneswar; All India Institute Of Medical Sciences - Bhubaneswar); Y Sakaray, S Irrinki, S Subbiah Nagaraj, S Khare, C Tandup (Chandigarh; Postgraduate Institute of Medical Education & Research, Chandigarh, India); M K B, AB Muthunayagam, A Prasath S V , P Arunachalam, P John (Coimbatore; PSG Institute of Medical Sciences and Research); S Raul, R Vakil, R Sinha, E Dvivedi, A Thomas, S Joseph, A Sharma, B Khan, D Chatterjee (DELHI; St Stephen’s Hospital); R Gupta, A Khanduri, S Singh, DH Tyagi , U Daspal, N Rawal, R Varshney (Dehradun; Synergy Institute of Medical Sciences); M Luthra, R Handa, S Basu , P Chadha, R Sethi (Delhi; Holy Family Hospital); T Longkumer, KK Mishra, S Sundaramurthy, S Kumar, D Phom, J Kaippally, D Ommi, L Imchen, T Alinger (Dimapur; Christian Institute of Health Sciences and Research); A Chhabra, A Kumar (Faridkot; Guru Gobind Singh Medical College & Hospital (Baba Farid University of Health Sciences)); B Kharga, M Sarda, K Bhutia (Gangtok; Sir Thutob Namgyal Memorial Hospital Sochakgang); N Sharma, MS Rodha, N Banerjee, A Baksi, S Kaur, R Chaudhary, M Lodha, SP Meena, M Badkur, I Singh, A Sinha, KJ Rathod, R Saxena, J Tk, A Vig, M Pathak, A Sukhdev Jadhav, S Nayak, T Motiwala, K Shreyas (Jodhpur; All India Institute of Medical Sciences (AIIMS), Jodhpur); SR Pathan, J Rathod, C Agarwal, K Sharma, S Pandya (Karamsad; Shree Krishna Hospital); A Anand, A Kumar, HS Pahwa, AA Sonkar, MK Agrawal, AK Pal (Lucknow; King George’s Medical University); D Jain, PD Haque, V Michael, W Bhatti, J Dhiman, DRS Thind, A Bhatt, P Gupta, A Luther, S Khurana, RR Ranadive, A Suroy, H Kaur, S D A, P Shukla (Ludhiana; Christian Medical College & Hospital); NK Chaudhry, DA Hajela, P Patel, PK Arya, DD Dhawan, DR Tripathi, KK Luthra, A Kumar, DH Gupta (Ludhiana; Satguru Partap Singh Hospital); A Mathew, C Pun, P Dummala, M Gurung (Madhepura; Madhepura Christian Hospital); P Alexander, N Aruldas (Manali; Lady Willingdon Hospital); PS Prabhu, S Payyanur Thotan, B L, B Sv (Manipal; Kasturba Medical College Hospital, Manipal); RD Sharma, R Redkar, R Nathani, S Karmarkar, A Sharma, S Singh, S Achugatla, A Bangar, DD Kulkarni, K Raghuwanshi, DN Nikam, SN Mahendra (Mumbai; Lilavati Hospital & Research Centre); B Sarang, D Belekar, K Gaikwad (Mumbai; Terna Medical College and Hospital); A John, PA Thomas, L Pramod, D Gavit, D Singh (Nandurbar; Chinchpada Christian Hospital); N Kansakar, N Gupta, N Kapur, N Narain (New Delhi; ABVIMS Dr RML Hospital); S Kulkarni (New Delhi; Army Hospital Research & Referral New Delhi); T Rashid, M Husain, F Tauheed, SV Manzoor , S Ohri (New Delhi; Hamdard Institute of Medical Sciences & Research); L Bains, P Lal, S Neogi, A Mishra, S Ahuja (New Delhi; Maulana Azad Medical College); T Iahmo, M George, M Singh , P Waghchoure, A Choudhrie (Padhar; Padhar Hospital); A Kumar, M Aggarwal, V Kanna D, S Vembar, TP Singh, DS Walia, V Singh, G Kaur, A Jindal, P Dhamija (Patiala; Government Medical College Patiala); M Kumar, A Sinha, AK Jha, M Sharma, A Bhadani (Patna; All India Institute of Medical Sciences, Patna); R Abhinaya, U Kumbhar, A Jain, S Chilaka , S P (Pondicherry; Jawaharlal Institute of Postgraduate Medical Education and Research); VS Jha, A Jayapalan, Y Vashishth, G Jalal, VV Nair, C Raphael , HK Prabhakar, Z Khan (Pune; Command Hospital, Southern Command); D Dugar, D Mohanty, TDB Tridip, DR Ramchandani (Raipur; All India Institute of Medical Sciences Raipur); S Basu, N Kumar, AG Goswami, H Panga, D Mallik, A Gupta, D Rajput, R Anjum T Siddeek, P Manjunath, S Edem, F Huda, SK Singh, S Karuppusamy Krishnasamy, S Katragadda, KMR Reddy, I Ahmed, E Yhoshu, L Manoj Joshua, A Das, P Kothari (Rishikesh; All India Institute Of Medical Sciences); K Singh , SS Malhi, R Kaur, HK Cheema, M Singh, N Saini, M Gupta, A Bhatti, A Gupta, P Kaur, N Pahuja , H Kaur, S Chopra, B Sehgal, G Singh (SAS Nagar (Mohali) ; BR Ambedkar State Institute of Medical Sciences Mohali); DS Kshirsagar, M Kaple, G Saxena, S Dhole, A Bhargava, C Mahakalkar, S Deshpande (Sawangi (Meghe), Wardha; Acharya Vinoba Bhave Rural Hospital); R Wani, RA Dar, AA Malik, N Bhat, ZA Shah, GA Bhat (Srinagar; Sher-i-Kashmir Institute of Medical Sciences); S Kondpan, A Kutma, JA Kalyanapu (Tezpur; Baptist Christian Hospital); A Sundaram, K J B, R Krishna raj, G George, M Chisthi, H Jafarkhan, I P s, U Govindan, MKL K S D, C Narayan, G Pillai, VV Kollengode, D Jabbar (Thiruvananthapuram; Government Medical College Thiruvananthapuram); T Tony V, A Nair, A Kavalakat, A Moncy, A Johnson, A Joseph, A Appukuttan, A J, AN Oommen, S Francis, S M, N Srinivas, M Narayanan (Thrissur; Jubilee Mission Medical College & Research Institute); T Devabalan Koil, MR Jesudason, Y Myla, ASP Dhinakar, A Tirkey, B Roopavathana. S, R Mittal, S Surendran, N Paul Ambrose, D Joshiba, P Trinity, R Raghunath, NP Paul Sigamony, PY George, R Philip Sridhar, S Chase, SJ Arthur (Vellore; Christian Medical College & Hospital).

Iran, Islamic Rep.: N Yousefzadeh Kandevani (Bastak; Farabi hospital); M Pourfridoni, H Askarpour, H Mohammadi sardoo, AA Kheirkhah Vakilabad, M Ali-Hassanzadeh (Jiroft; Imam Khomeini Hospital).

Iraq: R Raheem Attallah Al_obaidy (Anbar; Heet General hospital); Z Alkhuzaie, S Salim , FRH Hassooni (Najaf; Al Batool private hospital); Y Zwain , HMA Oneizah, S Razaq, M Razaq, HHZ .zaini (Najaf; Al-Najaf Al-Ashraf Teaching Hospital).

Jordan: S Alananzeh, S Al Momani, M Tanashat, O Altobaishat, S Alsmadi, N Al RABADI, L Sweidan (Ajloun; Al Iman Hospital); Z Alnajjar, G Alsheikh, N Mosleh, H Alzuhd, R Alkhatib, H Al-Abdallat, M Aljarawn, T Aloqaili, I Nadi, A Abdllah, O Al-Fahel, R Khalil, M Said, A Qasem, H Al-Fahel, M Hijazi, R Rabah, A Alaqtash, DB Badwan (Amman; Al-Basheer Hospital); Y Alawneh (Amman; Ibn Al Haitham Hospital); B Alrayes, M Salah, M Al-Qannas, H Abu Obead, I Alnimer, Y Alawneh, M Almaletti (Amman; Islamic Hospital); A Khamees, R Yousef Yassin, A Alsheikh, K Al-Shami, E Abu Siam, O Sarhan, KA Sawaftah, MAM Sawaftah, MA Sawaftah, M Sabri Massadi, Z Al-sheikh ali, N Raiq, O Ibrahim, J Al Karmi, M Diab, I Aburumman , AA Altawaiha , M Hasan, A AlZu’bi, L Yasin, B Yacoub (Amman; Jordan University Hospital); R Abu Salah, M Alqedrh , S Abu khousa, MEH Albanna (Amman; Marka Specialty Hospital); R Hussam Yacoub Hattar, D Samardali, R Refaie, L Hijazein , S Hammad, M Barbarawi, S Mamduh, J Al Daradkah, S Samardali , T Alshawabkeh (Amman; Prince Hamza hospital); M Mahafdah, Q Sabbah, SAM Ba-Shammakh, A Al Hammoud, M Bani hani, M Tabaza, H Malkawi, H Haj Freej, D Kasasbeh, B Dweik, K Ayyoub, OR Mahafdah (Ar Ramtha; King Abdullah University Hospital/ Jordan University of Science and Technology); R Hiary, I Shehadeh, L Dyab, R Daradkeh, R Raddad, R Alzu’bi, T Alhaj Hasan , B Alzoubi, M Alsharayri, M Nofal, S Ellouzy, M Al-Masri, S Fakhouri, N Absy, R Suleiman, O Mansour, JS Hadidi (As-Salt; Al Hussain New Salt Hospital); A Al-fandi (Irbid; Ar Ramtha Govermental Hospital); M Al-Fraijat, N Rabai, A Al-Zubeidy, R Abd Elkareem, S Bani Amer , T Alhusban, S AL-Doghme, R Abd Alkareem, R Damseh, M Alshami , T Majed, S Al Sharie, M Araydah, R Jaba’Teh, F Haddad, O Almomani, L M Mheidat, R Haddad, S Bataineh, S Ababneh (Irbid; Princess Basma Hospital).

Kazakhstan: M Kulimbet, N Maulenov, N Lakhanov, M Ramazanov, A Kiyabayev (Almaty; City Clinical Hospital No.7, Asfendiyarov Kazakh National Medical University); D Amangaliyev, A Shamsutdinova, A Polatbekov (Almaty; JSC ‘Central Clinical Hospital’, Asfendiyarov Kazakh National Medical University).

Kenya: R Parker, E Irungu, A Fadipe, F Ondago, G Waiyaki (Bomet; Tenwek Hospital).

Lebanon: A Khoneisser, A Kachi, B Abboud (Beirut; Hopital Libanais Geitaoui); M Chaccour, G Bechara, N Eshak, R Hleyhel, M Barakat (Jbail ; Maritime Hospital).

Libya: N Lindi, BH Hameed, A Abaidalla, N Mosbah, M Saleh khatab (Albayda; Albayda Medical Center); MAM Elghriani, A Ali, A Fathi, S Qwyder, M Saleh (Benghazi; Al-jalaa Teaching/Trauma Hospital); M Alshamikh, A Alhammali, A Aldurssi, A Gusibat, M Suleman, S Alashhab, M Denini, F Alowjaly, MM Almihashhish, F Elkhafeefi , H Altawati (Benghazi; Benghazi Children’s Hospital); S Elfallah, F Benghalbon, R Michael , M Abosedra, A Ahmayda, H Mftah (Benghazi; Benghazi Medical Center); M Bohlala, M Muragi, S Alneihuom (Darna; Al-Wahda Hospital); A Alkaseek, A Alshiteewi, H Shames, H Bileid Bakeer (Gharyan; Gharyan Central Hospital); N Albahloul, M Abudabbous, A Belkhair, A Abdelmalik, M Assalhi, M Altajouri, A Alailesh , A Alshukre (Misurata; Misurata Central Hospital); B Alazabi, M Alazabi, G Birqeeq, AAY Almugaddami, A Egdeer (Nalut; Nalut Central Hospital); H Embarek, M Bilfaqirah (Sebha; Al-Majd Clinic); M Abdelkabir, S Abdeewi , A Abdalhadi, M Benghazi (Sebha; Aseel Alghad Clinic); H Idheiraj, M Yahmad, M Alfaid, M Abdu, E Abdu, M Khalifa, G Matroud, A Amaigl , H Aldare, E Ali, K Shwail, K Abdulrahman (Sebha; Sabha Medical Center); A Bouhuwaish, A Emran, A Abdraba (Tobruk; Tobruk Medical Center); AE Elzoubi, A Belaid (Tripoli; Alkhalil hospital); A Alragheai, D Omar, S Magrhi, H Farhat, S Alsuwiyah , S Abrayik, S Bensalem, B Algettawi (Tripoli; Metiga Hospital); S Egreara, TMA Abdulmola, I Kandil (Tripoli; Sabratha teaching hospital); F Alshreef, F Elhabishi , M Alsori, L Shawesh , S Timmalah, M Alnuwayli, A Alhamadi, K Ahmed Ibrahim, S Abdullateef, R Altayargh, A Essamei, S Altoume, A Haidar, M Khalil, A Abdulnabi, S Mohammed , A Ghummied (Tripoli; Tripoli Medical Center/ Tripoli University Hospital); E Younes, S Elfurdag , A Ali, S Ashini, M Edeeb, Z Al-azher El-hamel, M Akkawe , N AlWAER, A Khair Etareig (Zawia; Zawia Teaching Hospital); B Allbakosh, H Abusnina, A Awidan, L Alokshi, M Iqreewi , M Almahjoub , H Altounsi, M Almaqrahi (Zliten; Zliten Teaching Hospital).

Madagascar: MJ Rakotonaivo, CF Rahantasoa Finaritra, JB Razafindrahita, YM Razafimandimby , A Rakotondrainibe (Antananarivo; Joseph Ravoahangy Andrianavalona Hospital).

Malaysia: AD Zakaria, MIS Ismail (Kelantan; Hospital Universiti Sains Malaysia); R Noor, M Che yaacob, SF Moh Pauzi, Z Chin (Kota Bharu; Hospital Raja Perempuan Zainab II); K Voon, JH Fu, JH Lim, SA Theivendran, NN Ramli (Kuching, Sarawak; Sarawak General Hospital); M Fitri, MA Yunus, AN Ramly, A Md Yunos, AAA Anuar (Malacca; Hospital Jasin); NS Abd Ghani, AAH Ahmad Zaidi, F Ashraf, M Mahadi, AA Abdul Rahim (Serdang; Hospital Pengajar Universiti Putra Malaysia (HPUPM)).

Mali: OAA Dicko, A Dembele, H Dolo, D Kone (Ségou; District Hospital of Tominian).

Mexico: TR Ibarra-Hurtado (Guadalajara; Antiguo Hospital Civil de Guadalajara); LA Flores Chávez, JA Flores Prado, K Jasso García, NE López Bernal, EV Romo Ascencio, LM Flores Chávez, MP Mellado Tellez, SA Ibarra Camargo, G Delgado Hernandez, JA Guzman Barba, LA Rea Bocanegra, M Tello Jimenez, JA Tavares Ortega, E Gómez Mejía, I Esparza Estrada, AA Salinas Barragan, JA Jimenez Flores, SJ Vázquez-Sánchez, J Gonzalez Garcia , ZM Correa López, FJ Barbosa Camacho (Guadalajara; Clínica de Especialidades más Centro de Cirugía Simplificada); CM Nuño-Guzmán, AM Nava Franco, JF Martinez Martin del Campo , JJ Ulloa Robles , L Bravo, ME Gonzalez-Gonzalez, FD Romo Rosales, TR Ibarra-Hurtado, LG Peña Balboa, C Yanowsky-Gonzalez, R Santana Ortiz, SA Trujillo Ponce, J Orozco-Perez, MDC Gonzalez, JE Gonzalez Aboytes, J Pizarro Lozano , JE Orozco Navarro, F Ibañez Ortiz , O Montaño Angeles, M Calderon, F Diaz , M Lazo Ramírez , JA Aguilar (Guadalajara; Hospital Civil Fray Antonio Alcalde); A Gonzalez Ojeda, C Fuentes Orozco, JM Chejfec-Ciociano, JM Carranza Rosales, MA Sánchez Audelo, CI Lupercio Figueroa, KV Ascencio Diaz, CE Gutierrez de la Rosa, F Mercado Sanchez, FY González Ponce, R Mares País, C González Baez, LÁ Pelayo Orozco, NG Barrera Lopez, A Ramírez Beas, MÁ Zaragoza Mendieta, MF Zarate casas , SL Trejo Ramos, P Salas Núñez, JA Gutiérrez Gómez (Guadalajara; Hospital de Especialidades, CMNO-IMSS); G Ambriz González, I Cabrera, HB Moya- Ambriz , FJ Silva Rivera, EM Torres De Anda, A Hernández, FJ León Frutos, VE Armenta Tapia, M Nieto Galvan, JM Alvarez Hernandez (Guadalajara; UMAE Hospital de Pediatria Centro Medico Nacional de Occidentes IMSS); AI Sánchez-Terán, N Muñoz Montes, AN Fuertes Muñoz, RL Smolinski kurek (León; Hospital Regional e Alta Especialidad del Bajio); K Bozada-Gutiérrez , A Nuñez Venzor, A Zubillaga-Mares, I Serrano (Mexico City; Hospital General Dr. Manuel Gea González); C Moreno-Licea, S Anaya Sanchez, A Trigos Díaz, CJ Pérez - Padrón, EY García-Villegas., RH Perez-Soto (Mexico City; Instituto Nacional de Ciencias Médicas y Nutrición ‘Salvador Zubirán’); MJ Rueda Medécigo, A Leon -del- Angel, HDJ Pérez Baca, C Chavarría Noya , L Castro (Pachuca; Sociedad Española de Beneficencia); D Herappe , MT Barrio Renteria (Querétaro; Hospital de especialidades del niño y la mujer); A Ramos-De la Medina, L Martinez, II Durán Sánchez , DS Gonzalez , MJ Martínez (Veracruz; Hospital Español Veracruz).

Morocco: M Berrakkouch, F Hourri, A Benmansour, R Ait Ben Addi , M Melouane, A Tariq, O Boujidi, I Zerrouq , M Katif, S Amahmid, S Errami, S Jamil, O Nouhail, H Essalim, A Nidali, N Ouachou (Marrakech; Centre Hospitalier Universitaire Mohammed VI, Marrakech); A Aboumedian , S Kessab, N Lahnaoui, O Arsalan, S Kassad, A Hrora (Rabat; Centre Hospitalier Universitaire Ibn Sina Rabat).

Namibia: JT Abebrese, M Van der Colf, FW Quayson , P Shimbulu, P Nambala (Windhoek; Windhoek Central Academic Hospital).

Nigeria: A Adeyeye, A Akinmade, E Afeikhena, AI Okunlola (Ado Ekiti; Afe Babalola University Multi-System Hospital); D Idowu, J Olorunfunmi, A Olabode (Ado-Ekiti; Ekiti State University Teaching Hospital); N Oloko, KJ Bwala, A Ningi (Bauchi; Abubakar Tafawa Balewa University Teaching Hospital Bauchi); P Agbonrofo, D Osifo , P Idjerhe , BO Izedomi, O Omoike, O Irowa, S Ideh, J Enaholo, C Agbonrofo , O Emuze , PV Odigie, M Ediale, RA Eghonghon, M Edena, A Ekpeti , M Momoh, C Osime, O Osagie, A Arekhandia, M Ibadin (Benin City; University of Benin Teaching Hospital); T AbdulRahman, R Ediru, J Abutu John, O Owolanke (Bida; Federal Medical Centre Bida); U Ezomike, N Agugua-Obianyo, J Ede, S Aliozor, EI Nwangwu, C Ilo, C Amah, L Onyebulu, I Ugwueke , I Obianyo, C Onwuzu, U Dilibe , I Orji, V Enemuo, N Celestine , N Ekwo (Enugu; University of Nigeria Teaching Hospital); SA Sani, S Olori, I Pius Ogolekwu , O Attawodi , R Hauwa SANI, P Chimezie Andrew (Gwagwalada; University of Abuja Teaching Hospital); A Ishola, O Ayandipo, N Akinbami, A Fakoya, TA Lawal, V Osoka, H Ogundipe (Ibadan; University College Hospital); A Ademuyiwa, F Alakaloko, O Oluseye , N Duru, M Ojo, T Olobatoke , AO Lawal, C Nwanmah , O Alaba, R Eloka, C Bode, O Elebute, J Seyi-Olajide, K Onyekachi, O Balogun, O Christianah, L Omomeji , F Akinwande, A Damola-Okesiji, J Okei (Idi Araba; Lagos University Teaching Hospital); H Abiyere, O Fatudimu, B Mustapha, O Babatunde, AI Okunlola (Ido Ekiti; Federal Teaching Hospital, Ido Ekiti); OM Williams, O Faboya, C Ónyeka, F Oni, K Shodunke, G Eke, M Abdulsalam , O Oso, A Ayodele, M Okechukwu (Ikeja; Lagos State University Teaching Hospital); C Adumah, A Talabi, O Oyinloye, O Olajide , V Agbakwuru, A Aderounmu, A Agbaje, YL Balogun, MO Ameen, DO Komolafe, O Olasehinde, O Ajiboye, M Fagbayimu, G Aduroja, O Fasoro, A Adisa, AM Olugbami, H Oyinlola, E Adebunmi (Ile-Ife; Obafemi Awolowo University Teaching Hospitals Complex); T Mohammed, A Lawal, F Bello, P Adebayo, O Salako, A Akinkuolie, MA Adetoyi, A Akeem Aderogba, T Oyeyemi , O Ojo (Ilesa; Obafemi Awolowo University Teaching Hospitals Complex Wesley Guild Hospital Unit); E Osaze, H Ekwuazi , I Ogundele, P Elemile, A Ayeni, I Okoro , C Onuoha (Ilishan-Remo; Babcock University Teaching Hospital); M Mobolaji-Ojibara , J Mohammad mohammad (Ilorin; General Hospital); NT Abdulraheem, A Jimoh , A Lawal, OK Fasiku (Ilorin; University of Ilorin Teaching Hospital); B Aminu , S Kache, G Yohanna Abrak (Kaduna; Barau Dikko Teaching Hospital); AA Sheshe, L Anyanwu, AB Muhammad, A Abubakar Abdulkarim , TN Nagwamutse, IU Garzali, S Muhammad, C Nwachukwu, IE Suleiman, M Abdullahi , SA Aji, A Dahiru, LB Abdullahi, SA Yunusa , A Yahaya, M Bello, I Wasiu, U Mohammed Bello, B Yunusa, N Umar (Kano; Aminu Kano Teaching Hospital); RE Enejo, N Nwafulume, A Oke, J Taiwo (Lokoja; Federal Teaching Hospital Lokoja); OH Ekwunife, OA Egwuonwu, OA Okoye, N Nwanne, C Ugwunne, J Ugwu, U Ezidiegwu, CD Nwosu, K Oluchukwu , V Modekwe, C Uche, EA Obiesie, C Osuigwe (Nnewi; Nnamdi Azikiwe University Teaching Hospital); OH Ekwunife, J Aseme, U Edith, J Ezeh, J Nnoli, H Willy-Chidire, D Chimkaomasiri (Onitsha; Holy Rosary Specialist Hospital); A Ojewuyi (Osogbo; UNIOSUN Teaching Hospital); I Ogundele, A Adekoya, L Amosu, A Oyedele, A Ayoade, BA Ayoade, A Asekun , A Ajayi, O Popoola, M Yinusa, AAA Oyelekan, O Oluyemi, C Nwosu, M Okudero, M Agunloye, A Ojo, C Nwokoro, I Babajimi-Joseph , A Williams, S Ogunlade (Sagamu; Olabisi Onabanjo University Teaching Hospital); M Daniyan, TT Sholadoye, M Bashir, N Oyelowo, SE Nwabuoku , A Yakubu, O Ogunsua, M Abubakar , MA Tolani, MS Aliyu (Zaria; Ahmadu Bello University Teaching Hospital).

North Macedonia: T Risteski, V Naunova, L Jovcheski (Skopje; University Clinic for Pediatric Surgery).

Pakistan: A Raza, A Hai, S Ahmed, DS Maqsood, S Chaudhary, M Farooq, M Tayyab, Z Qureshi, M Ali, S Maqbool, U Abdullah, M Aziz, A Irshad, S Said, A Zafar, U Akram, I Sadiq, A Abbas, M Siddique , I Shahbaz (Islamabad; Dr Akbar Niazi Teaching Hospital); SH Waqar, M Raheem, F Akhtar, DM Mehmood, DN Mahmood (Islamabad; The Pakistan Institute of Medical Sciences); M Shahid, A Ali, M Ahmed, M Mansoor Iqbal, Y Lakdawala, S Otho, M Khalid, M Masood, R Kumar, S Jabeen (Karachi; PAF Faisal Hospital); S Altaf (Karachi; Patel Hospital); M Abdullah, G Shamsi, I Ahmed , N Lodhi, G Awais, L Rai (Karachi; The Indus Hospital); S Khattak, A Janjua, A Liaquat, M Saleem (Lahore; Bahria International Hospital, Bahria Orchard); MN Rafique , MM Bin Khalid, H Ahmad, MS Khalid, MH Sadiq, A Hashmi , HH Shahid, MA Sadiq, MH Chishti, M Usama, M Kashif, AM Choudhary , H Basharat, K Khalid, MA Haider, MA Bashir, H Sabir, MF Tarar, M Usama (Lahore; Services Hospital Lahore); MB Mirza, WU Rehman, W Tahir, R Khalid, CE Azmat (Lahore; The Children’s Hospital & The Institute of Child Health Lahore); H Qayum, H Irfan Khan, A Mustafa (Peshawar; Mercy Teaching Hospital); HW Bhatti, MR Farooqui, F Rauf, NA Malik (Rawalpindi; Benazir Bhutto Hospital); M Usman Malik, S Hayat, D Riazhussain (Sargodha; District Headquarter & Teaching Hospital - Sargodha).

Palestine: J Najajra, N Al-Hroub, M Abu Daoud, R Jubran, M Srour, M Taamreh, A Alsalahat, H Masalma (Bethlehem, West Bank; Beit Jala Governmental Hospital (Al Hussein)); A Alwali, A Alwali, L Mohammed, M Al zebda, S Mahdi, A Shaheen, G Alrayyes, L Tafesh, T M. Abubasheer, M Abu Jayyab, H Jaber, M Abuwarda, B M. J. Alhaj, T Aldirawi, F Mahmoud, M Ali, A Albhaisi, M Obaid, WJN Almadhoun, A Alroobi (Gaza; Al-Shifa Hospital); M Abo Abdo, A Abuthaher, A AbuNemer, M Abu Al Amrain, I Nasser, A Awad, A AlAgha, R Madi, D AbuNemer (Gaza; Nasser Hospital); N Kullab, H Elhallaq, A Abu Tair (Gaza; Palestine Red Crescent Society - Al-amal Hospital); H Ayesh (Hebron, West Bank; Al-Ahli Hospital); H Abu-Arish, A Zamareh, M Ahmoud, M H. Oweidat, I AlJada, M Anati, S Halabi, W Alhroub, A Abuhammad, S M. Udwan, H Yaghmour (Hebron, West Bank; Governmental Hebron Hospital-Alia); D Houmran , NA Awwad, M Abed, I HajMohammed, A Hewari, A Alqerem, E Zidan, H Abbadi, S Abed (Jenin, West Bank; The Martyr Dr. Khalil Sulaiman Hospital (Jenin Governmental Hospital)); M Shakhshir, M HajHamad , M Saifi, S Abuzahra, A Khouli, Z Shabello, Z Khraim , S Ismail, MF Dwikat (Nablus, West Bank; Rafidia Hospital); R Bassam, A Sabbah, A Gharib, R Alzughayyar , R Issa, A Abuhantash, O Matar, Y A. Omar, O Khalil, A Awwad (Tulkarm, West Bank; Martyr Thabet Thabet Govermental Hospital).

Paraguay: A Rodriguez Gonzalez, ED Sosa Ferreira, R Ferreira Acosta , MN Martínez Bareiro, JE Giubi Bobeda, A Franco (Asuncion; Hospital de Clínicas, II Cátedra de Clínica Quirúrgica, Universidad Nacional de Asunción).

Peru: A Walfor (Huánuco; Hospital Regional Hermilio Valdizán Medrano); L Poggi, L Poggi, L Fuentes Rivera Lau, MA Moreno Gonzales, F Camacho, O Ibarra, G Arredondo (Lima; British American Hospital); K Nieto Yrigoin, D Chavez Fernandez, DC Juan Carlos (Lima; Clinica Internacional); G Mendiola, A Salazar, R Casma Bustamante (Lima; Hospital Santa Rosa de Lima); G Borda-Luque, Y Carpio Colmenares, MR Li Valencia, F Palomino Escalante, K Quispe de la Roca (Lima; SANNA - Clínica El Golf); MM Caramantin Obando (Paita; Hospital I Miguel Cruzado Vera EsSalud); R Polo, V Serna-Alarcon (Piura; Jose Cayetano Heredia III Regional Hospital).

Russian Federation: V Kakotkin, M Agapov, V Budyakova, S Dos Santos Rocha Ferreira, R Senin (Kaliningrad; Immanuel Kant Baltic Federal University, Regional Clinical Hospital); A Bedzhanyan, A Sumbaev, K Petrenko, E Bedzhanyan, E Tyurina, R Azimov, P Glushkov, K Shemyatovsky , S Husanov, A Sidorova (Moscow; Petrovsky National Research Centre of Surgery); G Yarovenko, E Shestakov, O Lisin, A Arustamyan, S Katorkin (Samara; Hospital Surgery Clinic of Samara State Medical University); J Sidorovskaia, K Cholah, I Cholah, D Kurochka (Simferopol; Municipal Emegency Hospital No.6); V Ten, Y Kudryavcev (Yuzhno-Sakhalinsk; Private healthcare institution ‘RZD-Medicine’).

Rwanda: JP Rugambwa, CN Nelly Rosine, N Jeannette, A Dusabimana (Huye, Gisagara; Butare university teaching hospital (CHUB)); C Seneza , C Uwakunda, L Mukamazera, G Ntwari, I Didier (Kigali; Kibagabaga Hospital); A Costas-Chavarri, M Eugene, C Nyampinga, R Munyaneza, D Muyenzi (Kigali; Rwanda Military Hospital).

Serbia: J Juloski, V Cuk, V Cijan, L Milic (Belgrade; Zvezdara University Medical Center).

South Africa: S Gumede, C Kloppers, K Booyse, S Dos Santos, M Flint (Cape Town; Groote Schuur Hospital); Z Johnson, JJ Jordaan, G Steenkamp (Cape Town; Karl Bremer Hospital); K Nieuwenhuys, J Uys, SS Verhage, A Goliath, S Gilbert (Cape Town; Khayelitsha District Hospital); M Kariem, N Karimbocus (Cape Town; Mitchell’s Plain District Hospital); C Lategan, T Mabogoane (Cape Town; Victoria Hospital Wynberg); S Mewa Kinoo, R Naidoo, N Ntanzi, S Sibiya, S Ebrahim (Durban; King Edward VIII Hospital); S Govender, E Naidoo, P Moodley, K Maharaj (Durban; Stanger Hospital); H Le Roux, J Van Niekerk (East London; Cecilia Makiwane Hospital); A Sparke, P Omwansa (East London; Frere Hospital); CA Baars, S Marawu, K Sevnaran, A Szpytko (Empangeni; Ngwelezana Hospital); G Charalambous, B Van Zyl, O Pheiffer, F Roodt (George; George Hospital); D Rattray, N Rasool, M Nkogatse , R Mackay, G Urdang (Johannesburg; Edenvale); V Manchev, D Clarke, S Naidu, V Govindasamy (Pietermaritzburg; Edendale Hospital); D Montwedi (Pretoria; Kalafong Academic Hospital); LC Kolongi , C Elliot-Wilson, S Kalenga , I Serfontein, M Goga (Upington; Dr Harry Surtie Hospital); S Burger, R Duvenage (Worcester; Worcester Provincial Hospital).

Sri Lanka: S Srishankar, SPB Thalgaspitiya, KJ Senanayake, D Wickramarathna (Anuradhapura; Teaching Hospital Anuradhapura); D Subasinghe, D Wickramasinghe (Colombo; National Hospital of Sri Lanka); M Nandasena, K Wijesinghe, H Miyasika, J Senavirathna, Y Chamara (Dehiwala; Colombo South Teaching Hospital); S Rajendra, SI Thuraisamy Sarma, B Balagobi, V Sutharshan , S Giridaran (Jaffna; Teaching Hospital, Jaffna); W Wijenayake, MT Ekanayake, S Jayatilleke, S Jayasekara, M Perera, R Perera, R Ellawala, WDD De Silva (Werahera; University Hospital, Kotelawala Defence University).

Sudan: S Alqurashi , N Rajab (Ed Dueim; Ed Dueim Teaching Hospital); TA Albushary , FA Mohammed Daoud , A Younis, MA Suliman, F Tahir Lwdie, S Ibrahim Tour Harakan, M Abdelhadi Suliman Adam , E Hegab, A Abdalla, T Almahdi , E Alabed (El Geneina; El Geneina teaching Hospital); M Ahmed, S Eldirdiri, M Salah, OA Eljizoly , A Mohammed, A Mohamed Ibrahim Mohamed (Gadarif city; Gadarif teaching hospital); A Albager, MA Ismael Alamin, M Alsalawi, MA Elgak (Kassala; Police hospital); EG Nubi Mohamed, A Eltahir, E Adel Hamdoun Aziz, I Adel, O Emadeldeen, OG Nubi, E Mohamed (Khartoum; Bashair Teaching Hospital); M Hamed, M Tageldin, E Elsheikh, U Omara, E ADAm, IMG Ahmed, GMG Ahmed, S Imam (Khartoum; Ibrahim Malik Teaching Hospital); AA Adam, S Amin Omar Alsiddig, A Ahmed, S Abdelrasoul Elnour Ismail, M Mohamedshafee, Y Mohamed (Khartoum; Omdurman Teaching Hospital); EE Abuobaida Banaga Hag El Tayeb, H Abuobaida , AS Ahmed (Khartoum; Ribat university hospital); A Elbalal, IMG Ahmed, GN El Hunjul, GMG Ahmed, A Mustafa, HA Fadlalmola (Khartoum; Soba University Hospital); A Mohammed, E Yousuf, E Hamed, S Ibrahim, O Morgan, N Omer (Wad Madani; University of Gezira Hospital).

Syrian Arab Republic: MA Farho, M Mohammad, AY Arnaout, Y Nerabani, Y Maktabi, W Alsado, A Anadani (Aleppo; Abd Al Wahab Agha Hospital); M Morjan, M Nasani, W Mayo, S Kreid, M Arnaout, MN Sawas (Aleppo; Al-Shahbaa Private Hospital); M Aloulou, MK Marawy, A Kezze, I Arnaout, A Kelzia, A Ghazal (Aleppo; Aleppo Private Hospital); A Ghazal, E Dabbagh, R Masri, MH Nabhan, A Alniemi, A Alhaj, S Ward, Y Haido, N Dadoush, MK Abu albahrain, A Niazi, W Abbas, A Hasan, S Alshab, S Kamari, R Kalouk, Z Toutounji (Aleppo; Aleppo University Hospital); D Sharl Ajami, B Alsaid, A Alusef, K Abo zaal (Damascus; Al assad university hospital); AR Hammadieh, Z Klib, MR Mslmani, A Alhaj zain, M Klib, L Shammas, AJ Chekfa, Z Odeh, R Joumaa, H Al-zoubi (Damascus; Al-Mouwasat University Hospital); Q Mashlah, HO Odah Bashi̇, H Zwaraa (Damascus; Children’s University Hospital); I Adham, L Hasan, A Khatib, S Jomaa, A Alfarwan, A Torbey, A Rashid, A Hawarah, A Ali, M Alhimyar, L Al-Boukhari, MA Al-yusuf, AN Aldirani (Damascus; Damascus Hospital); Y Alhammoud , Y Al-Junaidi (Homs; Al-Basel Specialized Hospital in Karm El-Louz); M Daher, Z Asaad, A Abbas, K Khalil (Homs; The Military Hospital); J Khoury, L Hasan, J Jahjah, J Alaji, S Turkmani, S Mahfoud, A Ahmad, M Derattani, G Massarra , J Skaff, ZA Hannouneh, H Amoudi, ZA Zaher, G Zaza (Latakia; Al Saydeh Surgical Hospital); A Wassouf, A Alahmad Alismael , S Nofal, A Mansour, M Mansour, Z Alkhaier, J Suliman, M Sabboh, M Haj Hussein, M Ibrahim, ZA Abo alaros, G Alhadwah , N Kheyrbek, D Ibrahim, G Hamdan, Y Hasan, A Abo al Shamat, A Roumieh, B Khattab, H Alkhatib (Latakia; National Hospital); S Hassan, S Abdul Rahman, A Abdul Rahman, F Aliskander, J Alkharish, G Kafa, I Suleiman, A Bassma, A Alloush , N Ismaiel, J Deeb, M Alrantisi, E Salloum, A Al-Mouahhed , A Baydoun, H Yunes, S Alkadi, F Ali, D Abdulrahman, I Hussein (Latakia; Othaman Hospital); A Bakri, H Asaad, T Ashkar, H Daaboul, A Marouf, F Chahrour, B Ranjous, B Ibrahim , A Sinjab, M Alneasan, N Ali, A Alloush, J Fahed, R Attaf, S Kanaan (Latakia; Tishreen University Hospital).

Thailand: A Tansawet, W Kasetsermwiriya, I Laopeamthong, P Sukhvibul, T Techapongsatorn, N Techapongsatorn, P Kasetsermwiriya , P Leungon (Bangkok; Vajira hospital).

Togo: PS Tekam Wadje (Lomé; CHU Sylvanus Olympio).

Tunisia: MJ Kacem, R Elaifia, Y Ouadi, S Megdiche, Y Jedidi (Tunis; La Rabta Hospital).

Turkey: AÇ Bozkurt, H Tümer (Adana; Adana Seyhan State Hospital); MA Koç, A Çakmak, AF Kocaay, KY Türker, TB Türkmen (Ankara; Ankara University Medical School); O Yalkın, D Yigit (Bursa; Bursa City Hospital); B Yigit, A Aslan, S Yılmaz (Elazig; Elazig Fethi Sekin City Hospital); AN Sanli, Yİ Tandoğan, A Yildiz, A İsler, A Ozkomec (Gaziantep; Abdulkadir Yuksel State Hospital); ME Seker, E Ay, M Erkaya, YO Koyluoglu, A Develioğlu, GM Kurtoglu, GK Kurtoglu, AF Cetişli, E Tunçcan, A Aghayeva, Z Durna, B Baca (Istanbul; Acibadem Altunizade Hospital); AE Dönmez, B Togay, E Ada, IE Yavuz , IA Bilgin, EC Karabulut, AM Uysal, Y Karataş, B Ağca, MK Aktas, F Demiral, B Duman, K Kabulov, I Hamzaoglu, T Karahasanoğlu, M Tanal, E Tuzuner (Istanbul; Acibadem Maslak Hospital); S Meriç, M Tokocin, H Yigitbas, A Barcin, G Alıcı, E Yavuz, OB Gülcicek, N Bugdayci , K Özdoğan, I Çakır, YE Aktimur, Y Altinel (Istanbul; Bagcilar Research And Training Hospital); ÖP Zanbak Mutlu (Istanbul; Bahçelievler State Hospital); RE Sönmez, M Şermet, MS Ozsoy, H Baysal, F Buyuker (Istanbul; Istanbul Medeniyet University, School of Medicine); M Oncel, S Bektas, AE Askin, M Yashar, A İzgi̇ş (Istanbul; Istanbul Medipol University Hospital); SS Uludağ, MF Ozcelik, S Yumurtacilar (Istanbul; Istanbul universty - Cerrahpaşa Medical faculty); A Özcan, E Somuncu, S Yilmaz, A Sapmaz, H Bolukbasi, C Özkan, E Bozdağ, MC Kizilkaya, H Telci, Y Kara, AZ Kaan, M Acar, EO Yildirim (Istanbul; Kanuni Sultan Suleyman Training and Research Hospital); G Akcakoca, C Hacıalioğlu, Y Tosun, V Çalik, TE Yılmaz, H Alfakeer (Istanbul; Kartal Dr. Lutfi Kirdar Training and Research Hospital); MI Ateş, SN Karahan, M Kalender, AE Narin, D Yi̇ği̇t, O Agcaoglu, S Toprak, B Celik, E Ozoran, DS Uymaz, S Yigman, E Bozkurt, A Rencuzogullari, E Balik, S Sucu (Istanbul; Koç University Medical School); A Akmercan, K Oğur, A Hajali, QK Dolatzay (Istanbul; Marmara University, School of Medicine); E Unal (Istanbul; Sehit Prof.Dr. İlhan Varank Training and Research Hospital); N Kiziltoprak, MS Genç, B Özcan, Z Şenol, OF Ozkan, M Çuhadar, ED Terzi (Istanbul; Sultan 2. Abdulhamid Han Training and Research Hospital, University of Health Sciences); T Gülşen (Istanbul; Sultanbeyli State Hospital); MT Demirpolat (Istanbul; University of Health Science Umraniye Education and Research Hospital); B Citgez, H Ozsahin, C Ersavas (Istanbul; Uskudar University Faculty of Medicine, Memorial Hospital); C Bi̇li̇r, AE Boztaş Demi̇r, AD Hacioglu, G Ozyuksel, H Ulman (Izmir; Bakircay University Cigli Training and Research Hospital); AM Öztürk, B Calik, AC Yaşar (Izmir; University of Health Sciences Izmir Bozyaka Training and Research Hospital); E Colak, MA Avci, E Aybar, M Gün, AB Ciftci, MS Uyanik, ME Kara, C Akgün, AC Sarı, Ö Küpçüoğlu, GO Kucuk, S Polat (Samsun; Samsun University Samsun Training and Research Hospital); N Kavak, MA Kara, G Karadeniz Cakmak, B Kum (Zonguldak; Zonguldak Bulent Ecevit University School of Medicine Research and Training Hospital); S Öztürk, B Eyduran (İzmir; University of Health Sciences Tepecik Training and Research Hospital).

Uganda: B Kigwe, M Arafat (Iganga; Iganga district hospital); M Nnabagulanyi (Kigumba; Kiryandongo Hospital); S Stonelake (Luwero; Kiwoko Hospital).

Yemen, Rep.: R Ghaleb, N Alnamari, A Al-Bahla, B Al soudi (Hajjah; Kowaydina hospital); B Alshaikh, M Al-Shehari, M Al-Dhaheri, YSSM Ghaleb, MI Issa (Sana’a; Al-Thawra Modern General Hospital).

**Section 2: Supplementary materials, tables and figures**

| **Supplementary materials** | **Pages** |
| --- | --- |
| **Supplementary tables title** | |
| Supplementary table 1: Measurement set mapped to WHO’s building blocks attributes. | 22 |
| Supplementary table 2: Hospitals characteristics across hospital types, based on the number of hospitals. | 23 |
| Supplementary table 3: Key performance measures to evaluate first referral hospitals. | 24 |
| Supplementary table 4: Postoperative complications at 30 days per hospital type. | 25 |
| Supplementary table 5: Additional descriptive measures to evaluate quality. | 26 |
| **Supplementary figure title** | |
| Supplementary figure 1: Map of included first referral hospitals in LMICs | 27 |
| Supplementary figure 2: Multilevel multivariable regression model testing factors associated with complications | 28 |

**Supplementary table 1: Measurement set mapped to WHO’s building blocks attributes for first referral hospitals.**

| **Attributes of WHO building blocks** | **Key performance measures** | **Additional descriptive measures** |
| --- | --- | --- |
| Access and coverage | Emergency rate | Waiting times |
|  | Financing methods | Bowel resection rate |
|  |  | Standardised patient pathways |
|  |  | Availability of day case surgical unit |
|  |  | Waiting list management |
| Quality and safety | Mesh use rate | Primary operator |
|  | Postoperative complications | Anaesthesia variation |
|  |  | Surgical approach variation |
|  |  | Day case rate |

**Supplementary table 2: Hospitals characteristics across hospital types, based on the number of hospitals.**

|  | **First referral hospital**  **(n=39)** | **Secondary level hospital**  **(n=83)** | **Tertiary level hospital**  **(n=206)** | **Total**  **(n=8,155)** |
| --- | --- | --- | --- | --- |
| **Hospital funding** |  |  |  |  |
| Public | 21 (53.8%) | 60 (72.3%) | 164 (79.6%) | 245 (74.7%) |
| Private | 15 (38.5%) | 21 (25.3%) | 35 (17.0%) | 71 (21.6%) |
| Public-Private | 3 (7.7%) | 2 (2.4%) | 7 (3.4%) | 12 (3.7%) |
| (Missing) | 0 | 0 | 0 | 0 |
| **Surgical emergency service** |  |  |  |  |
| Yes - surgery available 24h | 28 (71.8%) | 73 (88.0%) | 194 (94.2%) | 295 (89.9%) |
| Yes - surgery only during daytime | 4 (10.3%) | 9 (10.8%) | 6 (2.9%) | 19 (5.8%) |
| Yes - for assessment only | 3 (7.7%) | 1 (1.2%) | 3 (1.5%) | 7 (2.1%) |
| No | 4 (10.3%) | 0 (0%) | 3 (1.5%) | 7 (2.1%) |
| (Missing) | 0 | 0 | 0 | 0 |

Data are n (%) unless stated otherwise. Denominators for each group are presented at the top row.

**Supplementary table 3: Key performance measures to evaluate first referral hospitals.**

|  | **First referral hospitals** | **Secondary level hospitals** | **Tertiary level hospitals** | **Total** |
| --- | --- | --- | --- | --- |
| **ACCESS AND COVERAGE** | | | | |
| **Total** | n=494 | n=1,184 | n=6,477 | n=8,155 |
| **Emergency surgery** |  |  |  |  |
| Yes | 52 (10.5%) | 130 (11.0%) | 556 (8.6%) | 738 (9.1%) |
| No | 442 (89.5%) | 1053 (89.0%) | 5920 (91.4%) | 7415 (90.9%) |
| (Missing) | 0 | 1 | 1 | 2 |
| **Financing methods** |  |  |  |  |
| Insurance by the government | 247 (50.0%) | 820 (69.3%) | 4412 (68.1%) | 5479 (67.2%) |
| Other insurance^1^ | 58 (11.7%) | 199 (16.8%) | 511 (7.9%) | 768 (9.4%) |
| Out of pocket | 155 (31.4%) | 111 (9.4%) | 1124 (17.4%) | 1390 (17.0%) |
| Other^2^ | 34 (6.9%) | 54 (4.6%) | 430 (6.6%) | 518 (6.4%) |
| (Missing) | 0 | 0 | 0 | 0 |
| **QUALITY AND SAFETY** | | | | |
| **Mesh use** |  |  |  |  |
| Yes | 253 (68.8%) | 711 (83.4%) | 4118 (90.3%) | 5082 (87.9%) |
| No | 115 (31.2%) | 142 (16.6%) | 441 (9.7%) | 698 (12.1%) |
| (Missing) | 0 | 0 | 0 | 0 |
| **Total** | n=351 | n=803 | n=4245 | n=5399 |
| **Postoperative complications** |  |  |  |  |
| Yes | 84 (17.0%) | 135 (11.4%) | 1002 (15.5%) | 1221 (15.0%) |
| No | 410 (83.0%) | 1048 (88.6%) | 5473 (84.5%) | 6931 (85.0%) |
| (Missing) | 0 | 1 | 2 | 3 |

The table shows the number of patients experiencing each key performance measure. The rates presented are unadjusted rates. Waiting times are presented with median and interquartile range.

^1^ *Other insurance* included insurance provided by the employer, insurance that the patients has privately arranged and paid for and insurance but unknown how it was arranged.

^2^ *Other* included external funds or grants awarded by charities and non-governmental organisations and other methods of payment not mentioned elsewhere.

**Supplementary table 4: Postoperative complications at 30 days per hospital type.**

|  | **First Referral hospitals**  **(n=494)** | **Secondary level hospitals**  **(n=1,184)** | **Tertiary level hospitals**  **(n=6,477)** | **Total**  **(n=8,155)** |
| --- | --- | --- | --- | --- |
| **Complications** | | | | |
| Clavien-Dindo 0 | 410 (83.0%) | 1048 (88.6%) | 5473 (84.5%) | 6931 (85.0%) |
| Clavien-Dindo I | 63 (12.8%) | 99 (8.4%) | 756 (11.7%) | 918 (11.3%) |
| Clavien-Dindo II | 14 (2.8%) | 24 (2.0%) | 181 (2.8%) | 219 (2.7%) |
| Clavien-Dindo IIIa | 3 (0.6%) | 7 (0.6%) | 34 (0.5%) | 44 (0.5%) |
| Clavien-Dindo IIIb | 3 (0.6%) | 2 (0.2%) | 17 (0.3%) | 22 (0.3%) |
| Clavien-Dindo IVa | 0 (0.0%) | 2 (0.2%) | 2 (0.0%) | 4 (0.0%) |
| Clavien-Dindo IVb | 0 (0.0%) | 0 (0.0%) | 1 (0.0%) | 1 (0.0%) |
| Clavien-Dindo V | 1 (0.2%) | 1 (0.1%) | 11 (0.2%) | 13 (0.2%) |
| (Missing) | 0 | 0 | 0 | 0 |

Data are n (%) unless stated otherwise. Complications were classified according to Clavien-Dindo which were defined in the protocol.

**Supplementary table 5: Additional descriptive measures to evaluate quality.**

|  | **First referral hospitals** | **Secondary level hospitals** | **Tertiary level hospitals** | **Total** |
| --- | --- | --- | --- | --- |
| **QUALITY AND SAFETY** | | | | |
| **Total** | n=494 | n=1,184 | n=6,477 | n=8,155 |
| **Primary operator** | | | | |
| Senior surgeon | 370 (74.9%) | 931 (78.7%) | 4208 (65.0%) | 5509 (67.6%) |
| Trainee surgeon | 98 (19.8%) | 217 (18.3%) | 2225 (34.4%) | 2540 (31.2%) |
| Non surgeon, medical practitioners | 26 (5.3%) | 35 (3.0%) | 43 (0.7%) | 104 (1.3%) |
| (Missing) | 0 | 0 | 0 | 0 |
| **Anaesthesia variation** | | | | |
| General | 211 (42.7%) | 530 (44.8%) | 3010 (46.5%) | 3751 (46.0%) |
| Spinal | 221 (44.7%) | 502 (42.4%) | 2897 (44.8%) | 3620 (44.4%) |
| Loco-regional | 62 (12.6%) | 151 (12.8%) | 565 (8.7%) | 778 (9.5%) |
| (Missing) | 0 | 1 | 5 | 6 |
| **Surgical approach variation** | | | | |
| Open | 448 (90.7%) | 996 (84.2%) | 5516 (85.2%) | 6960 (85.4%) |
| MIS / MIS converted | 46 (9.3%) | 187 (15.8%) | 959 (14.8%) | 1192 (14.6%) |
| (Missing) | 0 | 1 | 2 | 3 |
| **Total** | n=351 | n=803 | n=4,245 | n=5,399 |
| **Day case adoption** | | | | |
| Yes | 129 (36.9%) | 343 (42.7%) | 1791 (42.2%) | 2263 (41.9%) |
| No | 221 (63.1%) | 460 (57.3%) | 2454 (57.8%) | 3135 (58.1%) |
| (Missing) | 1 | 0 | 0 | 1 |

Data are n (%) unless stated otherwise. Denominators for each group are presented at the top row.

MIS: minimally invasive surgery.

**
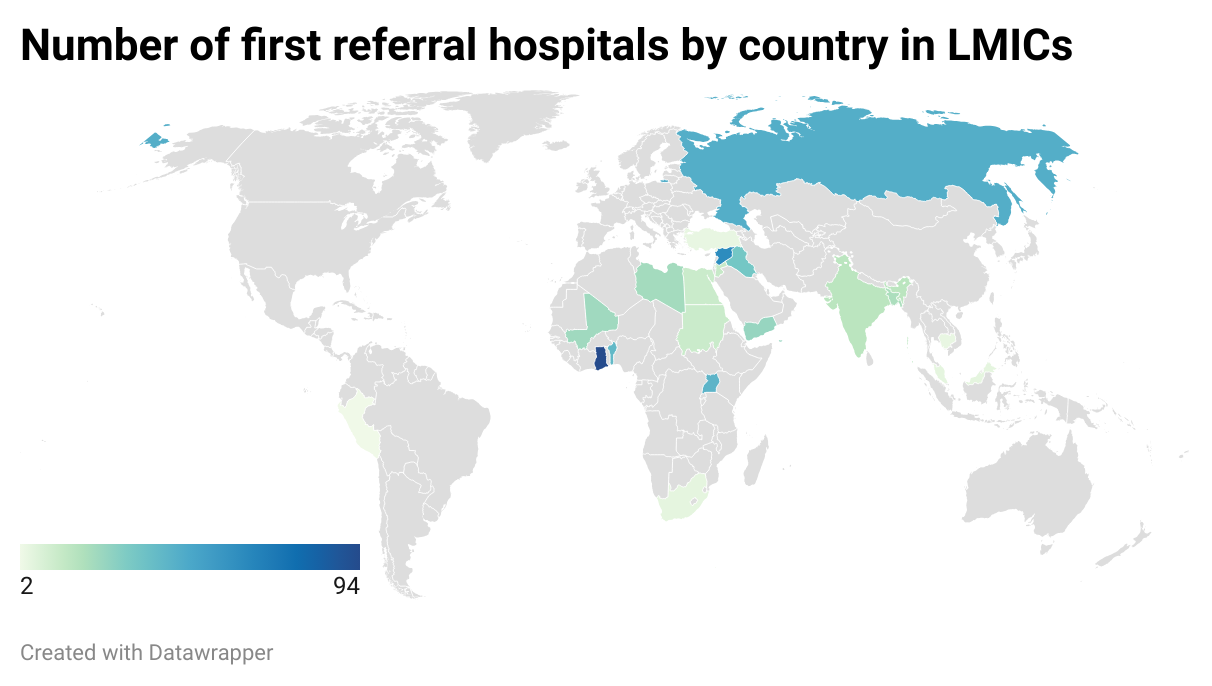
Supplementary figure 1: Map of included first referral hospitals in LMICs**

This map shows the countries where first referral hospitals were located. The colour gradient is proportionate to the number of included patients from these hospitals.

**Supplementary figure 2: Multilevel multivariable regression model testing factors associated with complications**

**
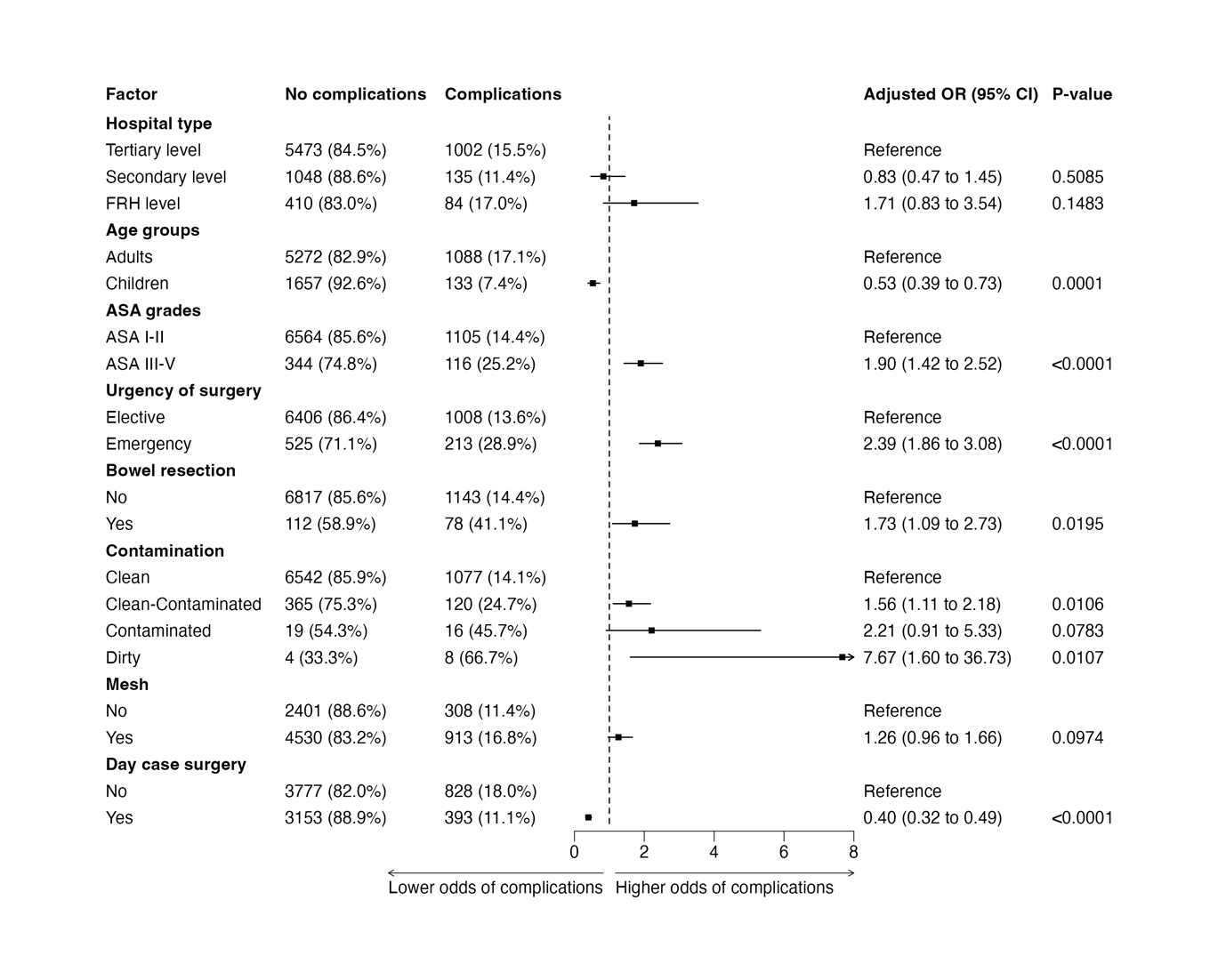
**

This model shows the factors associated with complications, reflecting a multilevel model with hospital and country as the two levels that were adjusted for. All patients were included in the model, except those with missing data for any of the variables (n=27). ASA: American Society of Anaesthesiologists Physical Status Classification System grade.
